# Cross-sectional associations of amyloid burden with semantic cognition in older adults without dementia: A systematic review and meta-analysis

**DOI:** 10.1016/j.mad.2020.111386

**Published:** 2020-10-19

**Authors:** Jet M.J. Vonk, Emma L. Twait, Rob J.P.M. Scholten, Mirjam I. Geerlings

**Affiliations:** a Department of Epidemiology, Julius Center for Health Sciences and Primary Care, University Medical Center Utrecht and Utrecht University, Utrecht, The Netherlands; b Department of Neurology, Taub Institute for Research on Alzheimer’s Disease and the Aging Brain, College of Physicians and Surgeons, Columbia University, New York, NY, USA

**Keywords:** Dementia, Preclinical, Prodromal, Non-demented, Neuropathology, Neuropsychology, Category fluency, Animal fluency, CSF, PET

## Abstract

Previous research suggests the presence of subtle semantic decline in early stages of Alzheimer’s disease. This study investigated associations between amyloid burden, a biomarker for Alzheimer’s disease, and tasks of semantic impairment in older individuals without dementia. A systematic search in MEDLINE, PsycINFO, and Embase yielded 3691 peer-reviewed articles excluding duplicates. After screening, 41 studies with overall 7495 participants were included in the meta-analysis and quality assessment. The overall weighted effect size of the association between larger amyloid burden and larger semantic impairment was 0.10 (95% CI [−0.03; 0.22], *p* = 0.128) for picture naming, 0.19 (95% CI [0.11; 0.27], *p <* 0.001) for semantic fluency, 0.15 (95% CI [−0.15; 0.45], *p* = 0.326) for vocabulary, and 0.10 (95% CI [−0.14; 0.35], *p* = 0.405; 2 studies) for WAIS Information. Risk of bias was highest regarding comparability, as effect sizes were often not calculated on covariate-adjusted statistics. The relevance of the indicated amyloid-related decline in semantic fluency for research and clinical applications is likely negligible due to the effect’s small magnitude. Future research should develop more sensitive metrics of semantic fluency to optimize its use for early detection of Alzheimer’s disease-related cognitive impairment.

## 1. Introduction

Alzheimer’s disease evolves from early pathophysiological changes in amyloid and tau proteins in the brain to manifest clinical dementia with overt cognitive and functional impairment across an estimated time span of 20 years or more (Bateman et al., 2012; Jansen et al., 2015; Villemagne et al., 2013). This process puts Alzheimer’s disease on a continuum of slow development, including a long preclinical phase in which the disease process evolves but no clinical diagnosis has been established yet. This preclinical phase is crucial for clinical trials aimed at intervention and for timely diagnosis for patients and caregivers.

The preclinical phase of Alzheimer’s disease is typically identified using biomarkers associated with the pathophysiological changes of the disease (Dubois et al., 2014). In the temporal evolution of Alzheimer’s disease biomarkers, amyloid-beta has been proposed to be among the first observable ones (Jack and Holtzman, 2013). Amyloid-beta can be detected using different methods, primarily by using histopathology, cerebrospinal fluid (CSF) assays, positron emission tomography (PET), or blood plasma assays. While Alzheimer’s disease biomarkers are often used in research, clinical application of these biomarkers including amyloid is less common due to considerations regarding expenses, invasiveness, and time to administer or evaluate (Zhang, 2019). Importantly, not everyone with elevated levels of brain amyloid develops dementia, and not everyone with clinically diagnosed Alzheimer’s disease has substantial amyloid burden (Brookmeyer and Abdalla, 2018; Glymour et al., 2018; Ossenkoppele et al., 2015).

Despite these discrepancies in the relationship between amyloid burden and development of dementia, the presence of amyloid in older adults without dementia has been consistently related to faster cognitive decline over time (e.g., Baker et al., 2017; Papp et al., 2016) and higher risk of incident Alzheimer’s disease (e.g., Chouraki et al., 2015; Koyama et al., 2012). In contrast to these associations over time with cognitive decline and future clinical endpoints, the cross-sectional relationship of amyloid burden with cognition in older adults without dementia is less clear across studies (Roostaei et al., 2017).

Tasks of episodic memory and semantic fluency (i.e., naming as many exemplars of a certain category as possible in a limited time) are the most reliable neuropsychological markers of progression to Alzheimer’s disease (Drago et al., 2011). Episodic memory is the most characteristic cognitive function impaired in early Alzheimer’s disease (Bäckman et al., 2001; Hodges, 1998). Additionally, a large body of research has demonstrated the role of subtle semantic decline in early stages of the disease or in individuals at high risk for Alzheimer’s disease (e.g., Chertkow et al., 2008; Papp et al., 2016; Vonk et al., 2019a). While a decline in episodic memory is not only present in Alzheimer’s disease but to a lesser extent also in normal aging, several aspects of semantic cognition stay relatively intact with normal aging (Horn and Cattell, 1966, 1967; Park et al., 2002; Salthouse, 2010; Vonk et al., 2020). Therefore, semantic cognition could play an important diagnostic and prognostic role in preclinical Alzheimer’s disease (Venneri et al., 2018).

A growing body of literature shows the presence of semantic memory impairment in preclinical AD and mild cognitive impairment (MCI) (Clark et al., 2009; Joubert et al., 2010; Murphy et al., 2006; Rao et al., 2015; Venneri et al., 2016), particularly in relation to amyloid burden (e.g., Papp et al., 2016, 2017; Snitz et al., 2013; Wirth et al., 2013). The pathogenic mechanisms that lead to the clinical manifestation of AD are still debated, including two recognized hypotheses: the amyloid cascade hypothesis (Hardy and Allsop, 1991) and the tau hyperphosphorylation hypothesis (Wischik et al., 1988). In the amyloid cascade hypothesis, in a complex pathway of events, amyloidosis leads to cognitive decline via synaptic dysfunctioning (amyloid plaques), and in the tau hyperphosphorylation hypothesis, in a complex pathway of events, extensive phosphorylation of tau leads to cognitive decline via microtubule dysfunctioning (neurofibrillary tangles) (Fan et al., 2019). The theoretical framework at the basis of the current study follows the amyloid cascade hypothesis. In both hypotheses, the dysfunctioning at the cell-level leads to neuronal loss in a network of brain regions, starting in the medial temporal lobe and subsequently spreading via the lateral temporal and parietal lobes to the frontal lobe (Dickerson et al., 2009). The brain regions affected early in the AD process, particularly the medial temporal lobe and temporal-parietal region, play a key role in semantic processing (Binder et al., 2009; Didic et al., 2011; Vonk et al., 2019b).

To date, two meta-analyses have investigated relationships between amyloid burden and cognition across multiple cognitive domains, including semantic memory, in older adults without dementia (Baker et al., 2017; Hedden et al., 2013). Both studies analyzed semantic cognition as a domain and included studies in their analyses that used a composite or factor score comprised of multiple tasks. However, there is a lot of variety in tests that are included in these domain scores, and some included tests may not reflect semantic processing. For example, Baker et al. (2017) classified letter fluency (i.e., naming as many exemplars starting with a certain letter as possible in a limited time) to represent semantic memory, while this task is generally thought to reflect executive functioning (Shao et al., 2014; Vonk et al., 2019b). Other semantic tasks, such as picture naming, typically suffer from a ceiling effect in healthy older adults (Moreno-Martínez and Laws, 2007). It is therefore important to consider these tasks separately in their relationship to amyloid burden to investigate subtle amyloid-related semantic impairment. This systematic review and meta-analysis investigated the presence and magnitude of associations between amyloid burden and semantic impairment across different types of semantic tasks in older individuals without dementia. We hypothesized that the increased power of a meta-analysis would reveal a small-to-moderate association between amyloid burden and performance on some but not all semantic tasks; we particularly expected an effect for the semantic fluency task, following previous findings on performance on this task among older individuals without dementia who were at higher risk for dementia (Papp et al., 2016; Vonk et al., 2019a).

## 2. Methods

The protocol for this systematic review and meta-analysis was registered in PROSPERO and is available in Supplementary Text 1. This systematic review and meta-analysis are reported following the PRISMA guidelines (Moher et al., 2009).

### 2.1. Search and selection

This systematic review and meta-analysis aimed to include all observational studies that reported on associations between amyloid burden and semantic cognition—measured within one year from each other—in older adults without dementia. We performed an electronic search on May 23, 2020 in MEDLINE (via the PubMed interface), PsycINFO, and Embase for peer-reviewed articles with no date or language restrictions. Unpublished materials, conference abstracts, and grey literature were not included.

The search string was developed for PubMed by adapting the search strings reported in Hedden et al. (2013) and Baker et al. (2017) in consultation with a librarian (PW, acknowledgments). The adjusted search string was tested for inclusion of the articles using semantic tasks in the meta-analyses by Hedden et al. (2013) and Baker et al. (2017), and subsequently translated to Embase and PsychINFO (Supplementary Text 2). Duplicates were removed using EndNote reference management software, and screening was performed using the Rayyan app (Ouzzani et al., 2016).

**Fig. 1.**
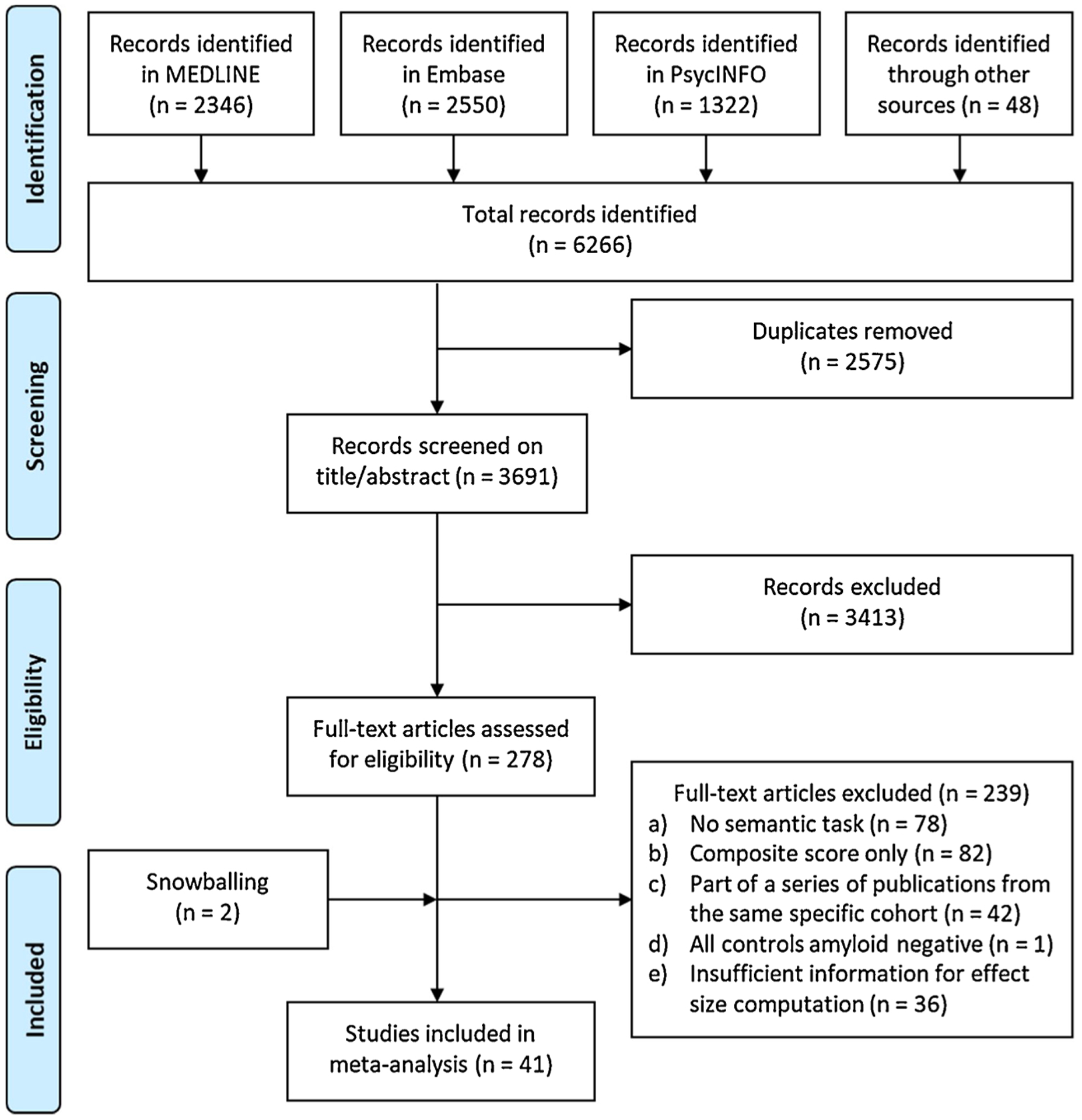
Flowchart study selection.

All studies that were identified in the search were first independently screened on title and abstract for inclusion based on eligibility criteria by two reviewers (JV and ET). The reviewers were blinded to each other’s decisions and resulting disagreements were resolved by discussion between the reviewers. Next, potentially suitable full texts as well as all studies using semantic tasks in the meta-analyses by Hedden et al. (2013) and Baker et al. (2017) were extracted and screened for eligibility. The reference lists from the selected studies were screened for additional articles (i.e., snowballing) and Scopus was used to screen articles that have subsequently cited the selected studies (i.e., reverse snowballing). The study selection processes were recorded using a PRISMA flow diagram (Fig. 1).

### 2.2. Eligibility criteria

A study was included if the study 1) reported associations between amyloid burden and semantic cognition, 2) measured amyloid and tasks of semantic cognition within one year from each other, 3) reported results for one or more separate semantic tasks, 4) reported results for nondemented older adults with an average age of *>*50 years, and 5) provided sufficient information in the publication for effect size computation.

Studies were excluded if they were limited to adults with an average age *<*50 years, individuals with all the same amyloid status (i.e., all amyloid positive or all amyloid negative), individuals with a diagnosis of MCI, and individuals with a diagnosis of neurodegenerative disease. Moreover, studies were excluded if they did not report results for individual semantic tasks but only reported results for a semantic, language, or executive function composite or domain score, if they did not reported amyloid status for the cognitively normal group, and if studies were published in another language than Dutch, English, German, or Farsi. If multiple studies reported results on the same task(s) from the same ongoing observational study, we included only the largest sample to represent the data for that semantic task(s) from that cohort to avoid introducing bias due to duplicate data.

### 2.3. Classification of determinant and outcome

The determinant of interest was amyloid burden, defined as either a continuous variable on a scale from no or low brain amyloid levels to high brain amyloid levels, or categorically as presence or absence of brain amyloid-positivity based on a cut-off value. Amyloid levels were determined using PET ligands, CSF or blood plasma assays, or histopathological evaluation.

The outcome of interest was performance on a semantic cognition task, including the following tasks: a) Boston Naming Test, Action Naming Test and other picture naming tasks or object naming tasks, b) semantic fluency, also called category fluency or animal fluency, and part of the Isaac Set Test, c) category verification task, d) synonym judgment task, e) WAIS Information, f) WAIS Similarities, g) WAIS Vocabulary or other vocabulary tasks, h) Pyramids and Palm Trees Test, Camel and Cactus Test, and other picture association tasks or word association tasks, i) word–picture matching. The outcome of interest was continuous, as the semantic tasks under consideration all had continuous outcome scores. We only considered performance on individual semantic tasks, not semantic domain or composite scores that included multiple different semantic tasks. We analyzed outcomes of semantic cognition performance in cross-sectional studies and scores at baseline in longitudinal studies, but not the rate of change from baseline to the last available follow-up in longitudinal studies.

### 2.4. Data extraction and risk of bias assessment

Information about study design and methodology, participant demographics and characteristics, amyloid method (PET, CSF, blood plasma, or histopathology) and metric (continuous or categorical), semantic cognition performance, and the associations between amyloid burden and semantic cognition tasks were extracted from the included studies and reviewed by two authors (JV and ET). If both unadjusted and adjusted effects were provided, preference was given to adjusted effects. If multiple methods were used, preference was given to methods in the following order: 1) histopathology, 2) CSF, 3) PET, and 4) blood plasma. If multiple categories of semantic fluency were reported separately, preference was given to semantic fluency of the category animals; not all cohorts use multiple categories, but typically all administer at least animals. Animals are the customary category because other categories are structurally biased by demographic factors, e.g., gender (Capitani et al., 1999; Marra et al., 2007).

Risk of bias in included studies was assessed at outcome-level using a modified version of the Newcastle-Ottawa Quality Assessment Scale Cohort Studies (Peterson et al., 2011). This modified scale using a star-based rating system consists of seven items to critically appraise a study on the quality of participant selection (maximum 3 stars), comparability of cohorts on the basis of the design or analysis (maximum 4 stars), and the quality of outcome assessment (maximum 2 stars) (Supplementary Text 3). Disagreements between judgments over the risk of bias in particular studies were resolved by discussion between the reviewers (JV and ET). If a sufficient number of studies (*>*10) was available for a semantic task, publication bias was assessed using funnel plots and by computing Egger’s t statistics.

### 2.5. Statistical analysis

Findings from the included studies were aggregated in overview tables and figures, and meta-analytically analyzed. All outcomes were transformed into effect sizes by using the studies’ reported statistics, e. g., mean and standard deviation or standard error, or results from analyses including t-tests, analysis of variance, correlations, regressions, and linear mixed-effects models. If available, values from analyses adjusted for age, sex, education, or potentially other variables were used. All effects were translated into standardized mean difference (Cohen’s *d*; for dichotomous measurements: mean difference/pooled standard deviations, for continuous measurements: standardized regression coefficients). If needed, the sign of effect sizes was changed so that positive effect sizes reflected greater amyloid burden associated with greater semantic impairment.

To obtain the pooled estimate for each semantic task, random-effects models with inverse variance weighting were used if a sufficient number of studies (5+) was identified. If only 2–4 studies were identified for a certain semantic task, we were bound to use a fixed-effects model with inverse variance weighting for methodological reasons, although results may be too optimistic when using a fixed-effects model. The models used a DerSimonian-Laird estimator for τ^2^. Differences in the association between amyloid burden and semantic cognition across the different semantic tasks were tested with subgroup analyses between tasks. We performed pre-specified subgroup analyses for amyloid assessment method (PET/CSF/blood/histopathology), use of a continuous versus categorical scale of amyloid burden, if a study did or did not control for demographic covariates (e.g., age, sex, education, or other), and if a study included only individuals with subjective cognitive complaints. We additionally performed posthoc subgroup analyses between participant samples with a mean age below versus above 70 years. A *p*-value below 0.05 was considered as a statistically significant result except for the Q-test (used in subgroup analyses), for which the threshold of statistical significance is typically set at *p* = .10 (Pereira et al., 2010).

Heterogeneity of the results was assessed using visual inspection of overlap in confidence intervals in the forest plot, Cochran’s Q test, and I-squared statistic. The amount of heterogeneity was interpreted according to the recommendations by the Cochrane Handbook (Higgins et al., 2019), i.e., 0–40% might not be important, 30–60% moderate heterogeneity, 50–90% substantial heterogeneity, and 75–100% considerable heterogeneity. The amount and impact of between-study variance was calculated using tau-square. The analyses and generation of figures (i.e., forest plots) were performed in R Version 3.6.0 (R Core Team, 2018).

## 3. Results

### 3.1. Search results and study characteristics

The search returned a total of 3691 studies after exclusion of duplicates. As detailed in the flow diagram in Fig. 1, screening yielded 41 studies for inclusion in the meta-analysis. The key characteristics of the included studies are presented in Table 1. The selected studies included a total of 7495 participants with a mean age ranging from 56.9–94.1, mean education (if reported in years) ranging from 9.3–17.3 years, and the proportion of female participants ranging from 39% to 74%. Of the 41 included studies, 21 (51.2%) reported on tasks of picture naming (3070 participants), 34 (82.9%) reported on tasks of semantic fluency (6753 participants), 2 (4.9%) reported on vocabulary (186 participants), 2 (4.9%) reported on WAIS Information (285 participants), and 1 (2.4%) on word-picture matching (118 participants). Amyloid-beta was analyzed as a continuous determinant in 9 (22.0%) studies. The method of amyloid assessment (for the majority of individuals in a study if multiple methods were used) was histopathology in 5 (12.2%) studies, CSF in 19 (46.3%) studies, PET in 15 (36.6%) studies, and blood plasma in 2 (4.9%) studies. In 29 (70.7%) studies, the sample size included 50 or more cognitively normal individuals. The provided information to calculate effect sizes was controlled for age, education, sex/gender, or other variables in 7 (17.1%) of the studies.

### 3.2. Risk of bias within and across studies

Quality assessment for risk of bias within the included studies is presented in Table 2. Studies scored between 3 and 9 stars, with 17 studies (41.5%) obtaining 5 or more stars. On selection criteria, studies lost stars because their sample was not representative of the average older adult without dementia in the community (16 studies, 39.0%) or because the categorization of amyloid was not based on established or published cut-offs (16 studies, 39.0%). On comparability, 38 studies (92.7%) lost stars because their effect sizes could not be calculated on information that was adjusted for age, sex/gender, education, and other covariates. All studies scored the maximum number of stars on the assessment of outcome.

**Fig. 2.**
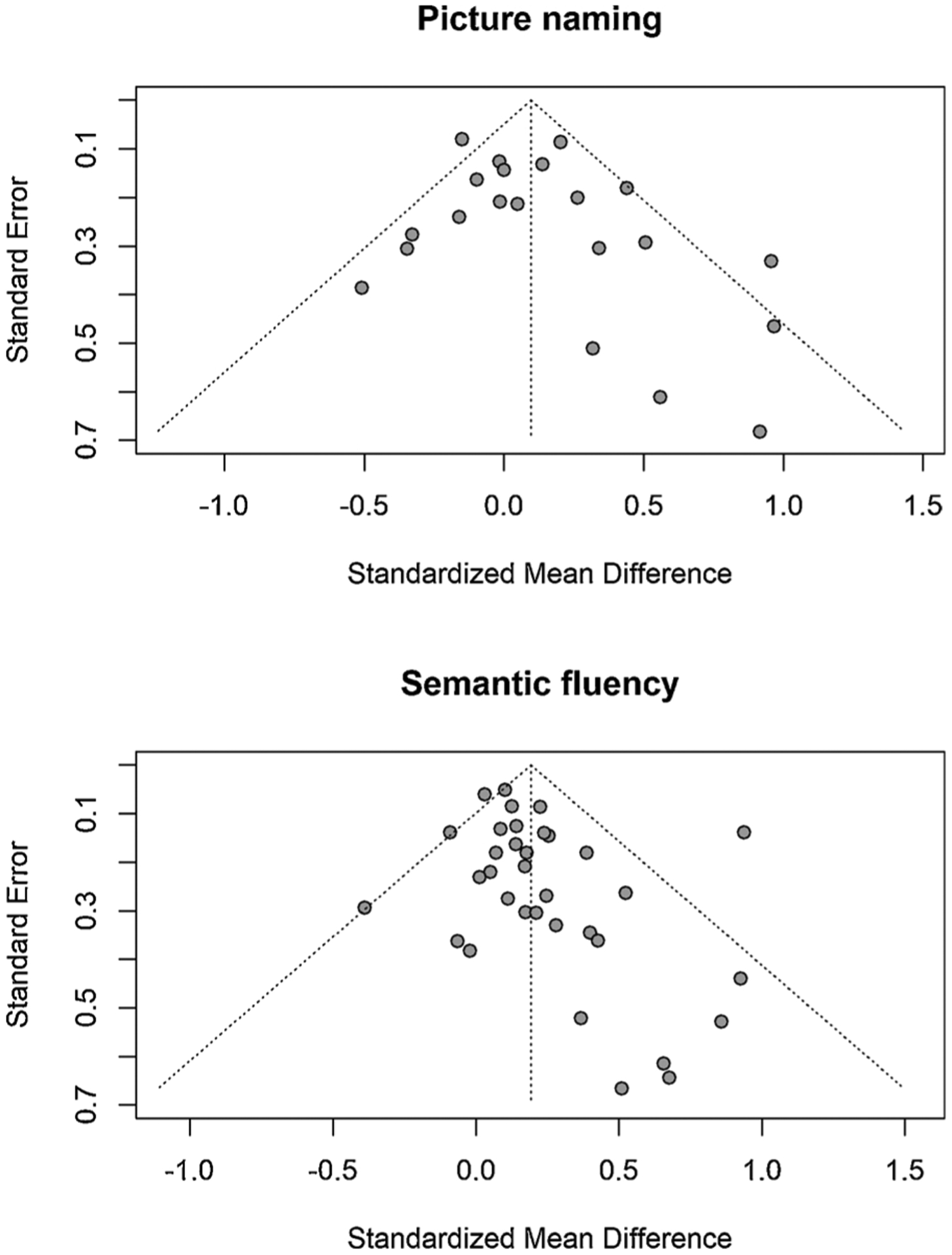
Funnel plots to assess publication bias.

Funnel plots for picture naming and semantic fluency to assess risk of bias across the included studies were not fully symmetric (Fig. 2). However, the Egger’s t statistic for asymmetry was non-significant for both picture naming (*b* for bias = 0.88, SE = 0. 59; *t*(19) = 1.509, *p* = 0.148) and semantic fluency (b for bias = 0.70, SE = 0.37; *t*(32) = 1.901, *p* = 0.066). For tasks of vocabulary, WAIS information, and word-picture matching not enough studies reported effects to assess publication bias for these tasks.

### 3.3. Meta-analysis

**Fig. 3.**
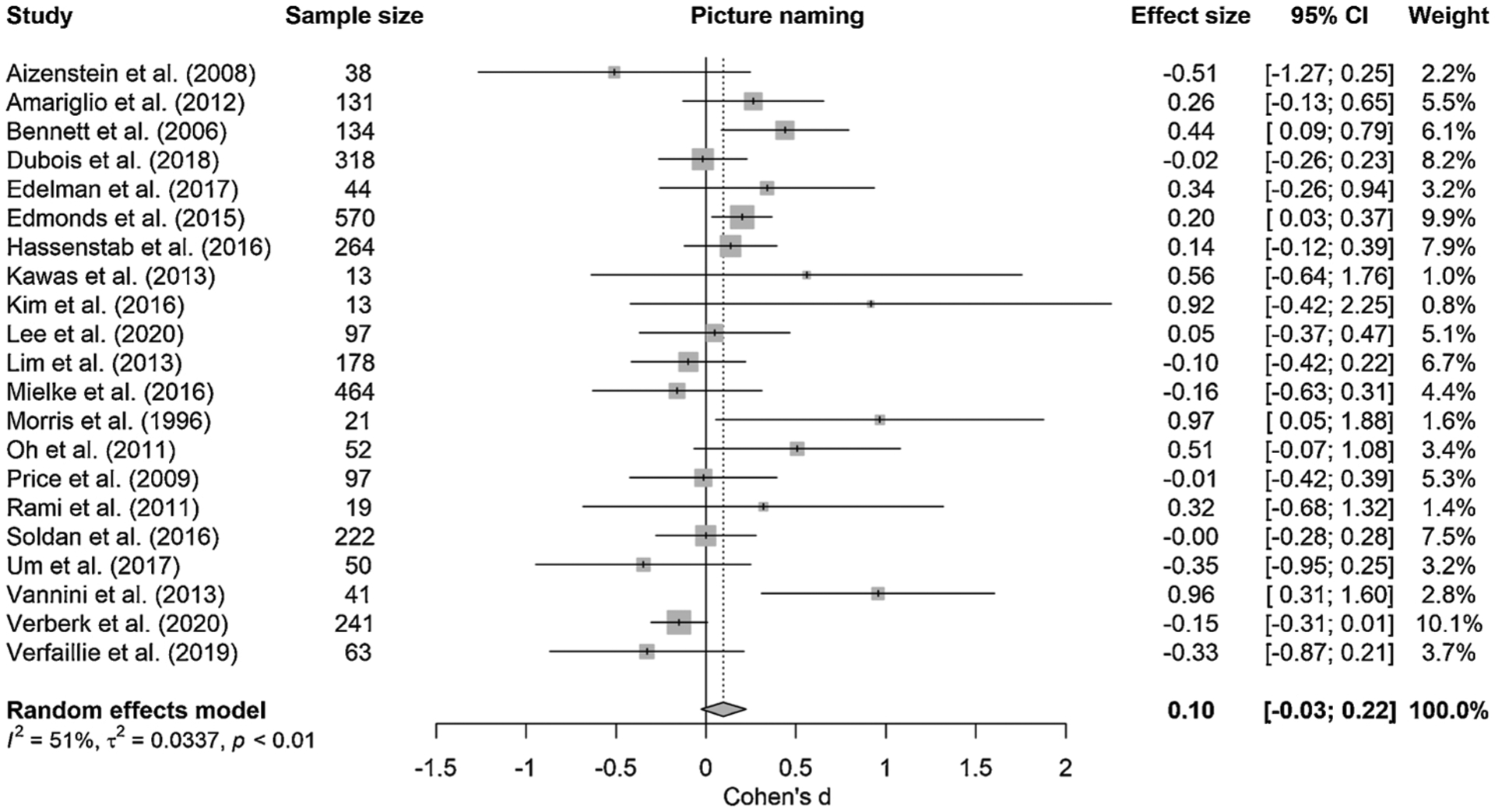
Forest plot of meta-analysis of the relationship between amyloid burden and picture naming; positive values represent greater impairment in performance in the presence of higher amyloid burden, dotted line represents no effect; size of the squares represents study weight.

**Fig. 4.**
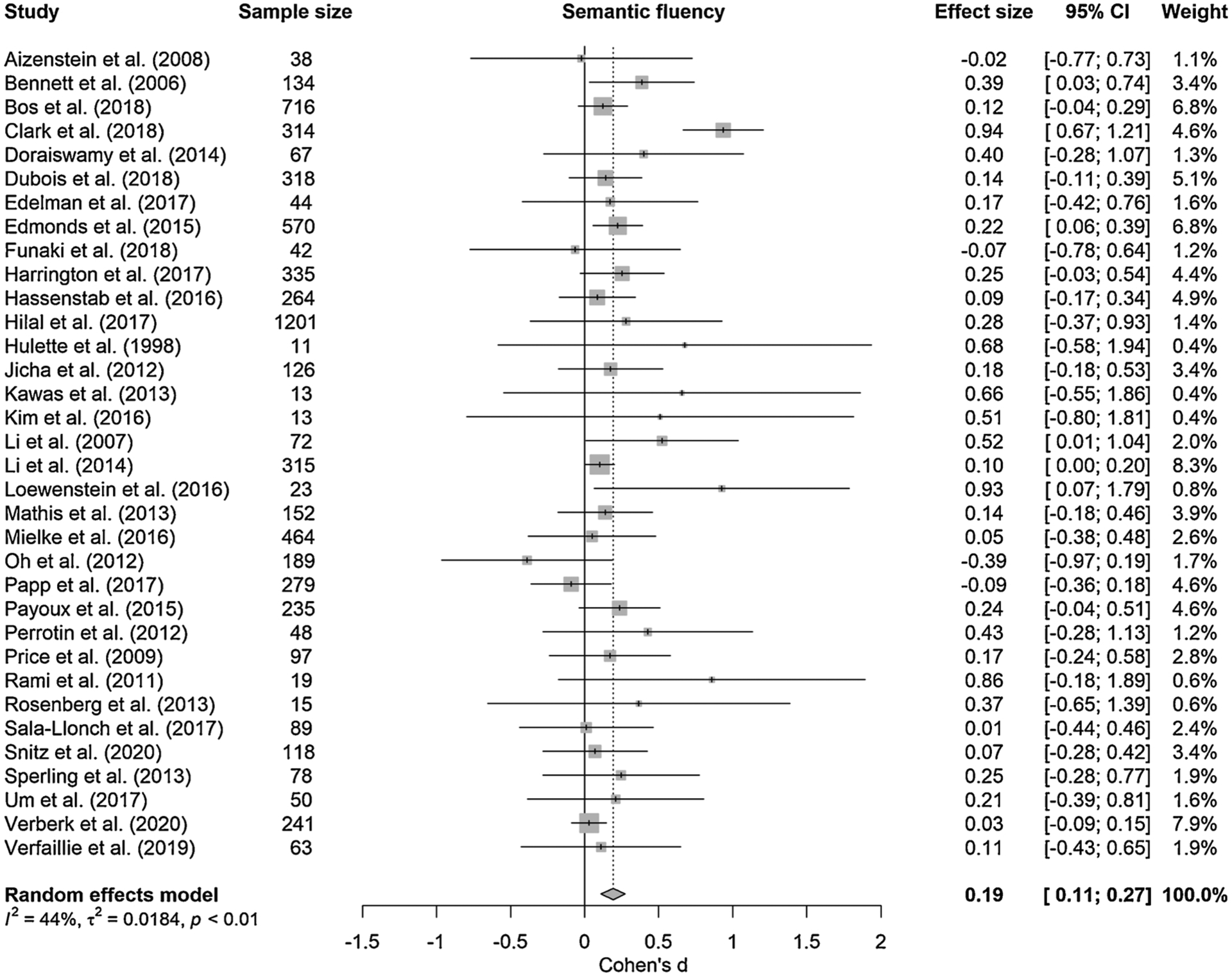
Forest plot of meta-analysis of the relationship between amyloid burden and semantic fluency; positive values represent greater impairment in performance in the presence of higher amyloid burden, dotted line represents no effect; size of the squares represents study weight.

**Fig. 5.**
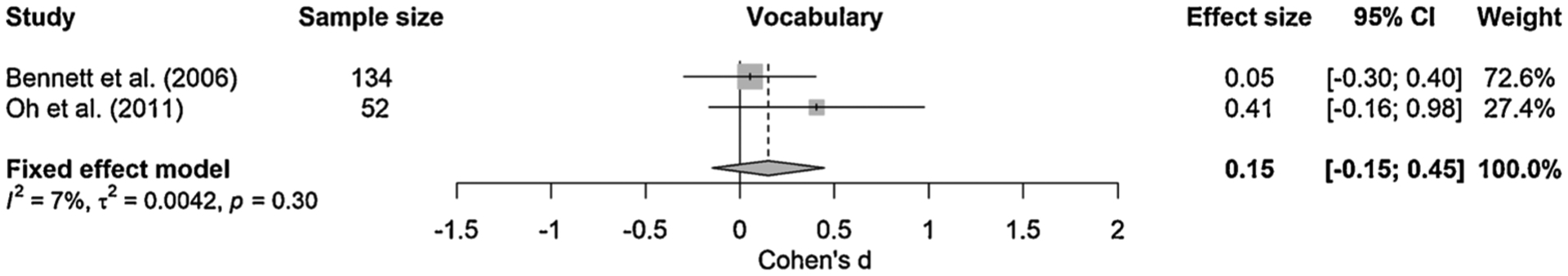
Forest plot of meta-analysis of the relationship between amyloid burden and vocabulary; positive values represent greater impairment in performance in the presence of higher amyloid burden, dotted line represents no effect; size of the squares represents study weight.

**Fig. 6.**
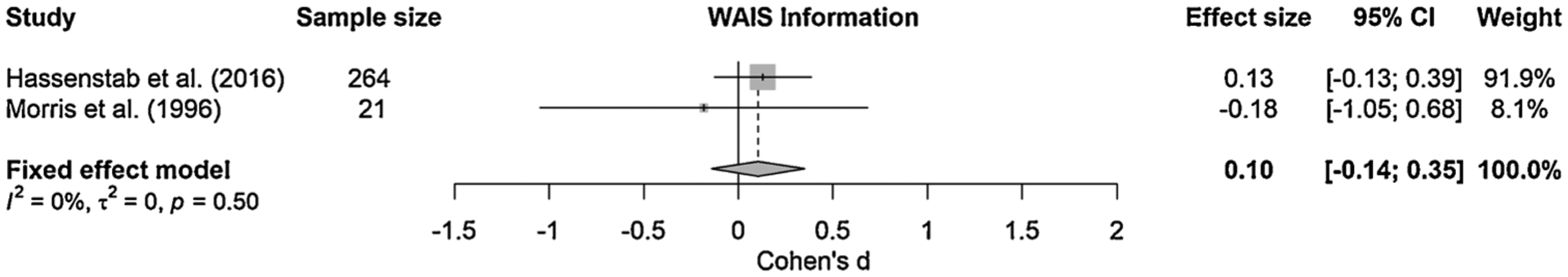
Forest plot of meta-analysis of the relationship between amyloid burden and WAIS Information; positive values represent greater impairment in performance in the presence of higher amyloid burden, dotted line represents no effect; size of the squares represents study weight.

Individual study effect sizes (Cohen’s *d*) for the four different semantic tasks that could be included in the meta-analysis, i.e., picture naming, semantic fluency, vocabulary, and WAIS Information are presented in Table 3. Figs. 3–6 show the forest plots per semantic task including effect sizes per study with 95% confidence intervals (CI) as well as the pooled results. The overall weighted effect size of the association between larger amyloid burden and larger semantic impairment was 0.10 (95% CI [−0.03; 0.22], *p* = 0.128) for picture naming, 0.19 (95% CI [0.11; 0.27], *p <* 0.001) for semantic fluency, 0.15 (95% CI [−0.15; 0.45], *p* = 0.326) for vocabulary, and 0.10 (95% CI [−0.14; 0.35], *p* = 0.405; 2 studies) for WAIS Information. These effect sizes are considered to be in the small-sized range, given that an effect size of *d* = 0.20 is considered small, *d* = 0.50 medium and *d* = 0.80 large (Cohen, 1988). Moderate heterogeneity in the pooled estimate of effect size was detected for picture naming (*Q* = 40.65, *p* = 0.004, I^2^ = 50.8%) and semantic fluency (*Q* = 59.00, *p* = 0.036, I^2^ = 44.1%), but not for vocabulary (*Q* = 1.07, *p* = 0.301, I^2^ = 6.7%) or WAIS Information (*Q* = 0.46, *p* = 0.499, I^2^ = 0.0%). For word-picture matching, which could not be meta-analyzed as only one study reported this task, the relationship with amyloid was −0.12 [−0.26; 0.02] (Snitz et al., 2020).

To investigate whether the association between amyloid burden and semantic cognition differed across tasks, we tested the difference between pairs of tasks with a subgroup analysis. We found no difference in pooled estimates across any pairs of tests: picture naming and semantic fluency (*Q* = 1.58, *p* = 0.208), picture naming and vocabulary (*Q* = 0.11, *p* = 0.736), picture naming and WAIS Information (= *<* 0.01, *p* = 0.956), semantic fluency and vocabulary (*Q* = 0.05, *p* = 0.821), semantic fluency and WAIS Information (*Q* = .44, *p* = 0.507), and vocabulary and WAIS Information (*Q* = 0.06, *p* = 0.805).

### 3.4. Subgroup analyses

We performed subgroup analyses for tasks of picture naming and semantic fluency, as the number of studies available for vocabulary (*n* = 2) and WAIS Information (*n* = 2) did not allow for stratified analyses.

For picture naming, the association with amyloid was stronger among studies with individuals who were not selected on having subjective complaints compared to studies that included only individuals with subjective complaints (15 vs. 6 studies; Q = 8.72, df = 1, *p* = 0.003), when the cohort’s mean age was above 70 years old compared to a mean cohort age below 70 years (14 vs. 7 studies; Q = 11.02, df = 1, *p* = 0.001), and when a study’s analysis did not control for any covariates compared to those that controlled for at least one covariate (18 vs. 3 studies; Q = 7.49, df = 1, *p* = 0.006). We observed no subgroup differences when stratifying the studies based on amyloid assessment method (*Q* = 2.12, df = 3, *p* = 0.547) or when an analysis used a categorical as opposed to a continuous value of amyloid (4 vs. 17 studies; *Q* = 2.09, df = 1, *p* = 0.148). Forest plots of these subgroup analyses, including more detailed results, are available in Supplementary Figs. 1–5.

For semantic fluency, the association with amyloid was stronger among studies with individuals who were not selected on having subjective complaints compared to studies that included only individuals with subjective complaints (29 vs. 5 studies; *Q* = 4.12, df = 1, *p* = 0.042), controlled versus uncontrolled for covariates (6 vs. 28 studies; *Q* = 4.87, df = 1, *p* = 0.027), and when an analysis used a categorical as opposed to a continuous value of amyloid burden (25 vs. 9 studies; *Q* = 4.07, df = 1, *p* = 0.044). We found no differences between subgroups when stratifying the studies based on amyloid assessment method (*Q* = 3.89, df = 3, *p* = 0.273), or if a cohort’s mean age was below versus above 70 years (12 vs. 22 studies; *Q* = 1.26, df = 1, *p* = 0.262). Forest plots of these subgroup analyses, including more detailed results, are available in Supplementary Figs. 6–10.

## 4. Discussion

This systematic review and meta-analysis summarized the evidence on the cross-sectional association between amyloid burden and semantic cognition in older adults without dementia. By pooling effect sizes of this relationship across multiple studies for separate tasks of semantic cognition, we increased the power and precision of the estimated effect size compared to individual studies. We found that higher amyloid burden was associated with more impairment in tasks of semantic fluency, but not picture naming, vocabulary, and WAIS Information. The magnitude of the effect of amyloid burden on semantic fluency was, following established conventions (Cohen, 1988), small. We detected moderate statistical heterogeneity signaling a certain inconsistency of effects across studies. Subgroup analyses showed that subjective cognitive impairment and covariate adjustment modified the effect of amyloid burden on both picture naming and semantic fluency, while age only modified the effect for picture naming and categorical/continuous amyloid scale only modified the effect for semantic fluency. Amyloid method (i.e., histopathology, CSF, PET, blood) did not modify the effect for either task. We did not find evidence for publication bias. Risk of bias within studies was highest with regard to comparability, as the majority of effect sizes could not be calculated on covariate-adjusted statistics.

The presence of a cross-sectional relationship between amyloid burden and semantic fluency differs from what is reported across both individual studies as well as meta-analyses. In the individual studies included in this meta-analysis, few studies reported a cross-sectional relationship between amyloid burden and semantic fluency. The lack of a relationship could be observed in the CIs of individual studies’ effect sizes, which nearly all contained the value zero, even though the vast majority of the studies reported a positive effect estimate between amyloid burden and semantic fluency impairment. This discrepancy between the pooled estimate versus individual study effects can be explained by the high variance in individual studies, which is reduced when the effect sizes are pooled across all studies due to increased power by enlarging the sample size.

The discrepancy at the meta-analytic level between this study and the pooled estimates by Hedden et al. (2013) and Baker et al. (2017) is likely not due to insufficient power to detect effects, since all three meta-analyses included at least 14 studies to calculate a pooled effect size for semantic cognition. Instead, the discrepancy is most probably due to the use of semantic domain scores in the analyses by Hedden et al. (2013) and Baker et al. (2017). Hedden et al. (2013) argued for the use of domain scores under the assumption that individual tests of a cognitive domain are similar in their representation of that domain. They noted, however, that this assumption is relatively difficult to test due to the wide variability in tests for certain domains. Because of the increase in the number of studies on the relationship between amyloid burden and cognition in recent years, we could test this assumption by investigating the relationship between amyloid burden and semantic cognition separately across different tasks. Our results demonstrated that not all semantic tasks are equally strongly related to amyloid burden. Thus, the assumption by Hedden et al. (2013) does not hold for the semantic domain, and combining these tasks in a domain score would dilute the presence of task-specific effects.

Differences in the magnitude of effect sizes across picture naming, semantic fluency, vocabulary, and WAIS Information may be caused for various reasons. Semantic cognition has multiple components, including semantic control, semantic memory efficiency, and semantic representation (Jefferies, 2013; Vonk et al., 2020; Whitney et al., 2011). Different semantic tasks may tap into these components with different weights, which may make the tasks differentially sensitive to early cognitive symptoms in the course of Alzheimer’s disease. Tasks of semantic cognition may also vary in their sensitivity to detect impairment in cognitively normal older adults due to limitations in their metrics. For example, picture naming typically suffers from a ceiling effect in cognitively normal individuals (Moreno-Martínez and Laws, 2007), since a task like the Boston Naming Test—the most popular picture naming task across studies—was developed for individuals with cognitive impairment due to aphasia (Kaplan et al., 1983).

While semantic fluency has no maximum score and no floor effect in older individuals without dementia, the traditional metric of total number of words generated may be too coarse to detect subtle semantic decline cross-sectionally at such an early stage of Alzheimer’s disease in individual studies. For example, the traditional metric of semantic fluency was not sensitive enough to distinguish amyloid-positive from amyloid-negative individuals at baseline but explained unique variance in amyloid-related decline over time (Papp et al., 2017). Moreover, the traditional metric of semantic fluency (total number of items) has been shown to fail at distinguishing non-demented APOE e4 carriers (i.e., genetic risk for Alzheimer’s disease) from non-carriers, while an alternative item-level metric was able to distinguish these groups (Vonk et al., 2019a). Since the current meta-analysis provided evidence for the presence of semantic impairment in the early preclinical phase, future research should focus on developing more sensitive metrics of semantic fluency or other sensitive semantic measures to be able to detect a larger effect in a smaller sample (Venneri et al., 2018, 2019; Vonk et al., 2019a).

Another explanation for the relatively weak association between amyloid burden and semantic cognition in this study, as well as with other cognitive domains in previous meta-analyses (Baker et al., 2017; Hedden et al., 2013), may be that the relationship between amyloid and cognition is mediated by tau pathology, following the tau hyperphosphorylation hypothesis. Increased amyloid may be a signal for increased tau pathology and associated neurodegeneration in semantic networks later in the pathway (e.g., Han et al., 2012; Hanseeuw et al., 2019). A recent study by Weigand et al. (2020) among 301 older adults without dementia from the Alzheimer’s Disease Neuroimaging Initiative (ADNI) contrasted cognitive performance across groups with and without amyloid and/or tau pathology. They showed that in the absence of tau pathology, no differences were found between amyloid-positive versus amyloid-negative individuals in memory, language, or executive functioning, while the largest effect sizes for all three domains were found when contrasting tau-positive versus tau-negative individuals in the amyloid-positive subgroup (Weigand et al., 2020). Another study showed that tau pathology, but not antecedent amyloid accumulation, correlated with cognition in individuals who were cognitively normal or had early symptoms of Alzheimer’s disease (Tosun et al., 2017). A literature review by Nelson et al. (2012) reported that at autopsy, the extent of cognitive impairment correlated more strongly with tau pathology than amyloid pathology. Thus, the specific impact of early Alzheimer’s disease on aspects of episodic memory and semantic cognition may be more strongly associated with tau pathology than amyloid pathology. Future research should explore differences in the strength of association among the three types of biomarkers of Alzheimer’s disease (i.e., amyloid, tau, and neurodegeneration) with episodic memory and semantic impairment for timely identification of individuals at high risk for clinical dementia.

Subgroup analyses contrasted various clinical and methodological factors of variability to explore sources of the moderate heterogeneity observed across effect estimates for both picture naming and semantic fluency. The relationship between amyloid and both tasks was stronger in studies that did not select only individuals with subjective impairment compared to studies that did. The etiology of subjective complaints can be highly heterogeneous (e.g., due to Alzheimer’s disease, other forms of dementia, depression, personality characteristics), particularly in a population that is different than one recruited in a memory clinic. More studies are needed to investigate the underlying cause of this difference across subgroups in relation to subjective cognitive impairment. The use of amyloid as a continuous versus categorical measurement revealed subgroup differences in semantic fluency, as the effect was weaker when amyloid was used as a continuous metric compared to a categorical metric. While not significant for picture naming, a similar pattern was observed in effect sizes between these subgroups. The use of amyloid as a continuous metric assumes a linear relationship with semantic impairment. Previous studies have shown that amyloid accumulation follows a sigmoid curve (Jack et al., 2013), with relatively slow accumulation at subthreshold biomarker levels followed by a relatively linear increase post-threshold until the accumulation rate levels off again at very high amyloid burden. Thus, using amyloid burden as a continuous metric may result in a weaker association between early amyloid burden and early semantic impairment due to this initial non-linear development of amyloid burden at subthreshold levels.

Several limitations of this research should be acknowledged, including various sources of bias. To avoid statistical bias by including the same individuals more than once, we had to exclude studies that reported results from the same cohort. We decided a priori to include the study with the largest sample size from a cohort. We should thus acknowledge that there are 42 more studies available that we could not include, but that have also investigated the association between amyloid burden and semantic impairment. We additionally had to exclude 36 studies that did not report sufficient information to compute an effect size of the association between amyloid burden and semantic cognition in cognitively normal older adults. We do not expect that exclusion of these studies substantially affected the pooled estimate of effect sizes, since we were able to include a substantial number of studies and we did not detect publication bias. However, additional inclusion of these studies—if sufficient information would have been reported—could have potentially reduced the uncertainty around the estimate, providing a more precise 95% CI. Future studies should adopt the standard practice of reporting effect sizes and confidence intervals with statistical estimates (Andrade, 2019), in addition to an extensive description of participant characteristics across all variables involved, including means and standard deviations across subsets of participants. Lastly, some of the subgroup analyses included relatively small groups of studies, which may have impacted the reliability of those results. Strengths of our study include the large number of included studies yielded by our thorough systematic search and the implementation of a quality assessment to outline the risk of bias within studies, which was not reported in either meta-analysis by Hedden et al. (2013) or Baker et al. (2017).

Assessment of cognitive abilities through neuropsychological testing is relatively easy, low-cost, and non-invasive compared to biomarker assessment—particularly in the context of primary care—and correlates with pathophysiological changes throughout the Alzheimer’s disease continuum (Baker et al., 2017; Hedden et al., 2013; Zhang, 2019). The results of this study confirmed the role of semantic impairment in early stages of Alzheimer’s disease. However, the relevance of the indicated amyloid-related decline in semantic fluency for research and clinical applications is likely negligible due to the effect’s small magnitude. Development of more sensitive semantic cognition markers of Alzheimer’s disease, in combination with biomarkers, could improve identification of high-risk individuals for early diagnosis and participation in clinical trials, and timely detection of Alzheimer’s disease-related symptoms in primary care settings.

## Supplementary Material

Supplementary Materials

## Figures and Tables

**Table 1:** Individual study characteristics.

	N	Cohort/sample origin	Language of administration	Age	Education (yr, or alternative)	Sex/gender
Study				m (SD, range)^1^	m (SD, range)^1^	woman
Aizenstein et al. (2008)	38	Pittsburgh community	English	74.4 (6.1)	15.1 (2.8)	63%
Amariglio et al. (2012)	131	Harvard Aging Brain Study (HABS)	English	73.5 (6)	16.1 (2.9)	53%
Bennett et al. (2006)	134	Religious Orders Study (ROS), Memory and Aging Project (MAP)	English	83.3 (6.4)	17.3 (4.3)	55%
Bos et al. (2018)	907	Barcelona St. Pau, EDAR, Gipuzkoa Alzheimer Project, Gothenburg MCI study, IDIBAPS, IMAP+, Leuven, EMIF preclinical-AD study, ADNI	Spanish, Swedish, Dutch, German, Greek, Danish	68.0 (9.1)	14.7 (3.7)	53%
Clark et al. (2018)	314	Wisconsin Alzheimer’s Disease Research Center (ADRC)	English	58.8 (37–85)	16.3 (2.5) (8–25)	69%
Doraiswamy et al. (2014)	67	AV45-A11	English	70.0 (11.3)	15.3 (2.2)	60%
Dubois et al. (2018)	318	INSIGHT-preAD Study	French	76.0 (3.5) (70–85)	68% ≥ HS	63%
Edelman et al. (2017)	44	Pittsburgh community	English	76.4 (5.6)	≥ 12 years	73%
Edmonds et al. (2015)	570	Alzheimer’s Disease Neuroimaging Initiative (ADNI)	English	73.1 (7.1)	16.4 (2.6)	46%
Funaki et al. (2019)	42	Keio University Hospital Memory Clinic	Japanese	74.4 (4.7)	15.1 (2.1)	48%
Harrington et al. (2017)	335	Australian Imaging, Biomarker, and Lifestyle Study (AIBL)	English	68.3 (5.7) (60–85)	Not specified	58%
Hassenstab et al. (2016)	264	Washington University Knight ADRC	English	72.1 (5.4) (65–86)	15.5 (2.8)	59%
Hilal et al. (2017)	1201	Rotterdam Study	Dutch	61.8 (7.2)	12.1 (4.0)	53%
Hulette et al. (1998)	11	University of Miami ADRC	English	81.6 (6.0)	16.6 (2.8)	50%
Jicha et al. (2012)	126	UK-ADC	English	83.7 (7.8)	16.0 (2.4)^2^	48%
Kawas et al. (2013)	13	The 90+ Study	English	94.1 (median) (90–99)	54% *>* HS	69%
Kim et al. (2016)	13	Yeungnam University Hospital in Daegu, Korea	Korean	72.0 (4.0)	9.5 (5.5)	54%
Lee et al. (2020)	97	Seoul, Korea	Korean	70.9 (7.5)	13.8 (3.6)	60%
Li et al. (2007)	72	Community University of Washington	English	67.9 (9.0)	15.7 (3.0)	54%
Li et al. (2014)	315	ADRC+	English	57.4 (18.1)	16.1 (2.6)	54%
Lim et al. (2013)	178	AIBL	English	71.6 (7.5)	Not specified	50%
Loewenstein et al. (2016)	23	University of Miami School of Medicine, Mount Sinai Medical Center, University of Florida	English	74.6 (8.1)	15.7 (2.9)	74%
Mathis et al. (2013)	152	Ginkgo Evaluation of Memory	English	85.5 (2.9)	14.8 (2.6)	42%
Mielke et al. (2016)	464	Mayo Clinic Study of Aging	English	62.7 (5.4) (50–69)	15.3 (2.3)	48%
Morris et al. (1996)	21	Washington University Faculty of Medicine	English	84.5 (6.6)	14.8 (3.3)	48%
Oh et al. (2011)	52	Community from Berkley	English	74.1 (6.0)	17.2 (1.9)	65%
Oh et al. (2012)	189	Community from Berkley	English	74.2 (6.0)	17.2 (2.0)	65%
Papp et al. (2017)	279	HABS	English	73.4 (6.0)	15.9 (3.0)	59%
Payoux et al. (2015)	235	Multidomain Alzheimer Preventive Trial (MAPT)	French	74.7 (4.3)	30% *>*12 yr	60%
Perrotin et al. (2012)	48	Berkley Aging Cohort Study	English	73.5 (5.9)	17.3 (1.9)	65%
Price et al. (2009)	97	National Alzheimer Coordinating Center (NACC)-supported Neuropsychological Database Initiative	English	84.3 (8.6)	15.4 (2.9)	57%
Rami et al. (2011)	17	Alzheimer’s Disease & Other Cognitive Disorders Unit of the Hospital Clinic in Barcelona	Spanish	68.7 (6.8)	9.3 (4.0)	N/A
Rosenberg et al. (2013)	15	John’s Hopkins ADRC, Joint Memory Clinics Baltimore, Memory Enhancement Center Eatontown, Community Health Center North East	English	74.5 (10.4)	17.3 (2.5)	39%
Sala-Llonch et al. (2017)	89	Oslo	Norwegian	73.1 (6.0) (65–90)	Not specified	48%
Snitz et al. (2020)	118	Monongahela-Youghiogheny Healthy Aging Team-Neuroimaging, Heart Strategies Concentrating on Risk Evaluation parent study	English	76.3 (5.7) (65–91)	74% *>* HS	58%
Soldan et al. (2016)	222	BIOCARD	English	56.9 (10.1) (22–85)	17.2 (2.3) (12–20)	60%
Sperling et al. (2013)	78	AV45-A05 study/24 different sites	English	69.4 (11.1)	15.2 (2.3)	56%
Um et al. (2017)	50	Catholic Geriatric Neuroimaging Database	Korean	67.5 (4.7)	9.6 (2.2)	64%
Vannini et al. (2013)	41	Brigham and Women’s Hospital and Massachusetts General Hospital	English	73.7 (0.5)	15.6 (0.5)	68%
Verberk et al. (2020)	241	SCIENCe and Amsterdam Dementia Cohort	Dutch	61.0 (9.0)	5.0 (1.0)^3^	40%
Verfaillie et al. (2019)	63	SCIENCe	Dutch	63.5 (8.2)	5.6 (1.3)^3^	43%

Note.

1 if available;

2 due to a typo in original paper the AB- group was reported to have an average of 60 years of education, thus we omitted this group in calculating mean years of education;

3 Dutch educational scale (Verhage, 1965);

*m* = mean, SD = standard deviation; cat = categorical, cont = continuous; PET = positron emission tomography, CSF = cerebrospinal fluid; yr = years, HS = high school.

**Table 2:** Newcastle-Ottawa scale for assessment of quality of included studies.

	Selection			Comparability				Outcome		Overall
Study	1. Representative	2. Selection	3. Exposure	Age	Sex/gender	Education	Other factors	1. Outcome	2. Same method	(max. 9)
Aizenstein et al. (2008)	✶	✶	–	–	–	–	–	✶	✶	4
Amariglio et al. (2012)	–	✶	–	–	–	–	–	✶	✶	3
Bennett et al. (2006)	–	✶	✶	–	–	–	–	✶	✶	4
Bos et al. (2018)	✶	✶	–	–	–	–	–	✶	✶	4
Clark et al. (2018)	✶	✶	–	–	–	–	–	✶	✶	4
Doraiswamy et al. (2014)	✶	✶	–	–	–	–	–	✶	✶	4
Dubois et al. (2018)	–	✶	✶	–	–	–	–	✶	✶	4
Edelman et al. (2017)	✶	✶	–	–	–	–	–	✶	✶	4
Edmonds et al. (2015)	✶	✶	✶	–	–	–	–	✶	✶	5
Funaki et al. (2019)	–	✶	–	–	–	–	–	✶	✶	3
Harrington et al. (2017)	–	✶	✶	✶	–	–	✶	✶	✶	6
Hassenstab et al. (2016)	–	✶	✶	–	–	–	–	✶	✶	4
Hilal et al. (2017)	✶	✶	✶	✶	✶	✶	✶	✶	✶	9
Hulette et al. (1998)	✶	✶	✶	–	–	–	–	✶	✶	5
Jicha et al. (2012)	✶	✶	✶	–	–	–	–	✶	✶	5
Kawas et al. (2013)	✶	✶	–	–	–	–	–	✶	✶	4
Kim et al. (2016)	–	✶	✶	–	–	–	–	✶	✶	4
Lee et al. (2020)	✶	✶	✶	✶	✶	✶	–	✶	✶	8
Li et al. (2007)	–	✶	✶	✶	✶	✶	✶	✶	✶	8
Li et al. (2014)	–	✶	–	–	–	–	–	✶	✶	3
Lim et al. (2013)	✶	✶	–	–	–	–	–	✶	✶	4
Loewenstein et al. (2016)	✶	✶	✶	–	–	–	–	✶	✶	5
Mathis et al. (2013)	✶	✶	–	–	–	–	–	✶	✶	4
Mielke et al. (2016)	–	✶	✶	✶	✶	✶	–	✶	✶	7
Morris et al. (1996)	✶	✶	✶	–	–	–	–	✶	✶	5
Oh et al. (2011)	✶	✶	–	–	–	–	–	✶	✶	4
Oh et al. (2012)	✶	✶	–	–	–	–	–	✶	✶	4
Papp et al. (2017)	✶	✶	✶	–	–	–	–	✶	✶	5
Payoux et al. (2015)	✶	✶	–	–	–	–	–	✶	✶	4
Perrotin et al. (2012)	✶	✶	✶	–	–	–	–	✶	✶	5
Price et al. (2009)	–	✶	✶	–	–	–	–	✶	✶	4
Rami et al. (2011)	–	✶	✶	–	–	–	–	✶	✶	4
Rosenberg et al. (2013)	✶	✶	✶	–	–	–	–	✶	✶	5
Sala-Llonch et al. (2017)	–	✶	✶	–	–	–	–	✶	✶	4
Snitz et al. (2020)	✶	✶	✶	✶	✶	✶	✶	✶	✶	9
Soldan et al. (2016)	–	✶	–	–	–	–	–	✶	✶	3
Sperling et al. (2013)	✶	✶	–	–	–	–	–	✶	✶	4
Um et al. (2017)	✶	✶	✶	–	–	–	–	✶	✶	5
Vannini et al. (2013)	✶	✶	✶	–	–	–	–	✶	✶	5
Verberk et al. (2020)	–	✶	✶	✶	✶	✶	–	✶	✶	7
Verfaillie et al. (2019)	–	✶	✶	–	–	–	–	✶	✶	4

*Note*. Each star represents if individual criterion within the subsection was fulfilled.

**Table 3:** Individual study’s analytic specifications and effect sizes with standard error.

Author	Total N	AB− n	AB + n	Amyloid scale	Amyloid method	SCD only	Covariate-controlled	Picture naming	Semantic fluency	Vocabulary	WAIS Information	Word-picture matching
Aizenstein et al. (2008)	38	29	9	categorical	PET	no	no	−0.51 (0.39)	−0.02 (0.38)	–	–	–
Amariglio et al. (2012)	131	97	34	categorical	PET	no	no	0.26 (0.2)	–	–	–	–
Bennett et al. (2006)	134	84	50	categorical	histology	no	no	0.44 (0.18)	0.39 (0.18)	0.05 (0.18)	–	–
Bos et al. (2018)	907	526	190	categorical	CSF or PET	no	no	–	0.12 (0.08)	–	–	–
Clark et al. (2018)	314	240	104	categorical	CSF	no	no	–	0.94 (0.14)	–	–	–
Doraiswamy et al. (2014)	67	57	10	categorical	PET	no	no	–	0.4 (0.34)	–	–	–
Dubois et al. (2018)	318	230	88	categorical	PET	yes	no	−0.02 (0.13)	0.14 (0.13)	–	–	–
Edelman et al. (2017)	44	23	21	categorical	PET	no	no	0.34 (0.3)	0.17 (0.3)	–	–	–
Edmonds et al. (2015)	570	142	428	categorical	CSF	no	no	0.2 (0.09)	0.22 (0.09)	–	–	–
Funaki et al. (2019)	42	32	10	categorical	PET	no	no	–	−0.07 (0.36)	–	–	–
Harrington et al. (2017)	335	277	58	categorical	PET	no	yes	–	0.25 (0.14)	–	–	–
Hassenstab et al. (2016)	264	177	87	categorical	CSF	no	no	0.14 (0.13)	0.09 (0.13)	–	0.13 (0.13)	–
Hilal et al. (2017)	1201	–	–	continuous	plasma	no	yes	–	0.28 (0.33)	–	–	–
Hulette et al. (1998)	11	7	4	categorical	histology	no	no	–	0.68 (0.64)	–	–	–
Jicha et al. (2012)	126	71	54	categorical	histology	no	no	–	0.18 (0.18)	–	–	–
Kawas et al. (2013)	13	9	4	Categorical	PET	no	no	0.56 (0.61)	0.66 (0.61)	–	–	–
Kim et al. (2016)	13	10	3	continuous	PET	yes^1^	no	0.92 (0.68)	0.51 (0.67)	–	–	–
Lee et al. (2020)	97	–	–	continuous	plasma	no	yes	0.05 (0.21)	–	–	–	–
Li et al. (2007)	72	51	21	categorical	CSF	no	no	–	0.52 (0.26)	–	–	–
Li et al. (2014)	315	–	–	continuous	CSF	no	yes	–	0.1 (0.05)	–	–	–
Lim et al. (2013)	178	123	55	categorical	PET	yes	no	−0.1 (0.16)	–	–	–	–
Loewenstein et al. (2016)	23	–	–	continuous	PET	no	no	–	0.93 (0.44)	–	–	–
Mathis et al. (2013)	152	74	78	categorical	PET	no	no	–	0.14 (0.16)	–	–	–
Mielke et al. (2016)	464	383	81	categorical	PET	no	yes	−0.16 (0.24)	0.05 (0.22)	–	–	–
Morris et al. (1996)	21	12	9	categorical	histology	no	no	0.97 (0.47)	–	–	−0.18 (0.44)	–
Oh et al. (2011)	52	33	19	categorical	PET	no	no	0.51 (0.29)	–	0.41 (0.29)	–	–
Oh et al. (2012)	189	34	18	categorical	PET	no	no	–	−0.39 (0.29)	–	–	–
Papp et al. (2017)	279	209	70	categorical	PET	no	no	–	−0.09 (0.14)	–	–	–
Payoux et al. (2015)	235	158	77	categorical	PET	no	no	–	0.24 (0.14)	–	–	–
Perrotin et al. (2012)	48	27	11	categorical	PET	no	no	–	0.43 (0.36)	–	–	–
Price et al. (2009)	97	59	38	categorical	histology	no	no	−0.01 (0.21)	0.17 (0.21)	–	–	–
Rami et al. (2011)	17	11	6	categorical	CSF	yes	no	0.32 (0.51)	0.86 (0.53)	–	–	–
Rosenberg et al. (2013)	15	–	–	continuous	PET	no	no	–	0.37 (0.52)	–	–	–
Sala-Llonch et al. (2017)	89	62	27	categorical	CSF	no	no	–	0.01 (0.23)	–	–	–
Snitz et al. (2020)	118	–	–	continuous	PET	no	yes	–	0.07 (0.18)	–	–	−0.12 (.07)
Soldan et al. (2016)	222	148	74	categorical	CSF	no	no	0 (0.14)	–	–	–	–
Sperling et al. (2013)	78	60	18	categorical	PET	no	no	–	0.25 (0.27)	–	–	–
Um et al. (2017)	50	34	16	categorical	PET	no	no	−0.35 (0.31)	0.21 (0.3)	–	–	–
Vannini et al. (2013)	41	22	19	categorical	PET	no	no	0.96 (0.33)	–	–	–	–
Verberk et al. (2020)	241	–	–	continuous	CSF	yes	yes	−0.15 (0.08)	0.03 (0.06)	–	–	–
Verfaillie et al. (2019)	63	44	19	categorical	PET or CSF	yes	no	−0.33 (0.28)	0.11 (0.27)	–	–	–

*Note*. SCD = subjective cognitive decline; PET = positron emission tomography, CSF = cerebrospinal fluid;

1 All subjects had a history of major depressive disorder in addition to SCD.
