# Supplementary material for: Cross-sectional associations of amyloid burden with semantic cognition in older adults without dementia: A systematic review and meta-analysis
Source: Mech Ageing Dev. Author manuscript; Available in PMC 2021 Mar 11. (PMC7952036; doi:10.1016/j.mad.2020.111386)

# Supplementary Materials

## Supplementary Text 1. Registered protocol (PROSPERO)

*1. Review title.*

A systematic review and meta-analysis of the association between amyloid burden and semantic cognition tasks in older adults without dementia

*2. Original language title.*

N/A

*3. Anticipated or actual start date.*

May 20, 2020

*4. Anticipated completion date.*

May 20, 2021

*5. Stage of review at time of this submission.*

The review has not yet started: Yes

| Review stage | Started | Completed |
| --- | --- | --- |
| Preliminary searches | No | No |
| Piloting of the study selection process | No | No |
| Formal screening of search results against eligibility criteria | No | No |
| Data extraction | No | No |
| Risk of bias (quality) assessment | No | No |
| Data analysis | No | No |

*6. Named contact.*

Dr. Jet Vonk

*7. Named contact email.*

J.M.J.Vonk-5@umcutrecht.nl

*8. Named contact address*

Julius Center, Epidemiology

Huispostnummer Stratenum 6.131

Postbus 85500 3508 GA Utrecht

*9. Named contact phone number.*

N/A

*10. Organisational affiliation of the review.*

Julius Center for Health Sciences and Primary Care, University Medical Center Utrecht, Utrecht, The Netherlands

https://juliuscentrum.umcutrecht.nl/en-US

*11. Review team members and their organisational affiliations.*

- Dr. Jet Vonk, Julius Center for Health Sciences and Primary Care, University Medical Center Utrecht, Utrecht, The Netherlands
- Emma Twait, Julius Center for Health Sciences and Primary Care, University Medical Center Utrecht, Utrecht, The Netherlands
- Dr. Mirjam Geerlings, Julius Center for Health Sciences and Primary Care, University Medical Center Utrecht, Utrecht, The Netherlands

*12. Funding sources/sponsors.*

- Alzheimer Nederland (WE.15-2018-05, PI: J.M.J. Vonk)
- ZonMw NWO Veni grant (project number 09150161810017, PI: J.M.J. Vonk)

*13. Conflicts of interest.*

All authors declare that they have no known conflicts of interest.

*14. Collaborators.*

N/A

*15. Review question.*

- What is the association between amyloid burden and various semantic cognition tasks and in older adults without dementia?
- Which task of semantic cognition shows the strongest association with amyloid burden?

*16. Searches.*

Three databases, i.e., Medline (via PubMed Interface), PsycInfo, and Embase, will be searched for peer reviewed articles with no date or language restrictions. All relevant citations from selected articles from the online database search will be consulted; snowballing and reverse snowballing will be performed using Scopus. Unpublished materials (or only abstracts) and grey literature will not be included.

*17. URL to search strategy.*

*18. Condition or domain being studied.*

Older adults without dementia.

*19. Participants/population.*

Inclusion:

- Studies that include older adults without objective cognitive impairment
- Studies that include a sample of adults with an average age of >50 years
- Studies that include participants who have been administered at least one test of semantic cognition
- Studies that include participants who have amyloid levels determined using PET/CSF/blood plasma/histopathology

Exclusion:

- Studies that include adolescents (under 18 years of age)
- Studies that include individuals with a diagnosis of mild cognitive impairment (MCI)
- Studies that include individuals with a diagnosis of neurodegenerative disease, including but not limited to all-cause dementia, Alzheimer’s disease, vascular dementia, frontotemporal dementia, Lewy Body dementia, Motor Neuron Disease, Creutzfeldt-Jakob Disease, Multiple System Atrophy, Normal Pressure Hydrocephalus, Parkinson Disease, Parkinson Disease Dementia, Posterior Cortical Atrophy and semantic dementia

*20. Intervention(s), exposure(s).*

The exposure to be reviewed is amyloid burden, defined as either on a scale from no or low brain amyloid levels to high brain amyloid levels (continuous variable), or as presence or absence of brain amyloid-positivity based on an established cut-off value (categorical variable).

Methods of assessing amyloid burden include:

- CSF or blood plasma assays
- PET ligands
- histopathology

*21. Comparator(s)/control.*

Semantic cognition in individuals with high levels of brain amyloid or individuals who are categorized as amyloid-positive is contrasted to semantic cognition in individuals with low levels of brain amyloid or individuals who are categorized as amyloid-negative, respectively.

*22. Types of study to be included.*

Inclusion:

- Observational studies that report associations between amyloid (measured via CSF or blood plasma, PET imaging, histopathology) and semantic cognition measured within one year from each other

Exclusion:

- Studies that do not report results for individual measures but only report results for a semantic or language composite/domain score combining performance on several semantic or language tasks
- Studies published in another language than Dutch, English, German, or Farsi
- Studies not published in peer-reviewed journals

*23. Context.*

N/A

*24. Main outcome(s).*

The outcome to be reviewed is performance on semantic cognition, which includes the following tasks and metrics:

- Boston Naming Test, Action Naming Test and other Picture Naming tasks or Object Naming tasks
- Word–picture matching
- Category fluency (also called semantic fluency or animal fluency, and part of the Isaac Set Test)
- Category verification task
- Synonym Judgment Task
- WAIS Information
- WAIS Similarities
- WAIS Vocabulary or other vocabulary tasks
- Pyramids and Palm Trees Test, Camel and Cactus Test and other Picture Association tasks or Word Association tasks

The outcome of interest is continuous (i.e., each semantic task has a continuous outcome score). We will only consider individual semantic tasks, not semantic domain composite scores. Outcomes of semantic cognition performance in cross-sectional studies and at baseline in longitudinal studies will be considered in this review (i.e., change from baseline to the last available follow-up will not be considered).

*25. Additional outcome(s).*

N/A

*26. Data extraction (selection and coding).*

Study selection: Two reviewers (JV and ET) will be applying eligibility criteria and selecting studies for inclusion in the systematic review. These reviewers will independently screen records for inclusion and will be blinded to each other’s decisions. Disagreements will be resolved by discussion among these reviewers. References will be managed in EndNote (e.g., duplicate removal). The software system for recording decisions will be Rayyan; the first step of screening will be on abstracts and titles, and subsequently potentially suitable full texts will be extracted and screened. Study selection processes will be recorded using a PRISMA flowchart.

Data extraction: Information about study design and methodology, participant demographics and baseline characteristics, amyloid method and metric, semantic cognition performance, and the associations between amyloid burden and semantic cognition tasks will be extracted from study documents. Two people (JV and ET) will independently extract data, which will be subsequently combined to ensure full coverage of data that should be obtained from the studies. Disagreements in data extraction will be resolved by discussion among the data extraction team (JV and ET). The extracted data will be recorded in Excel.

*27. Risk of bias (quality) assessment.*

Two people (JV and ET) will independently assess the risk of bias in included studies (i.e., at study-level) using the Newcastle - Ottawa Quality Assessment Scale Cohort Studies. The quality assessment will be performed independently by JV and ET, while blinded from each other’s assessment. Disagreements between reviewers’ judgements over the risk of bias in particular studies will be resolved by discussion among these reviewers. If a sufficient number of studies is available (>10), publication bias will be assessed using funnel plots.

*28. Strategy for data synthesis.*

Findings from the included studies will be aggregated in an overview table and figures, and meta-analytically analyzed. If multiple studies report estimates on the same tasks from the same ongoing observational study, we will include only the largest sample to represent the data for that/those semantic task(s) from that cohort.

All outcomes will be transformed into effect sizes by using the studies’ reported statistics (e.g., mean and standard deviation or standard error, or results from analyses including t-tests, analysis of variance, correlations, regressions, and linear mixed effects models). If available, values from adjusted analyses will be used (adjusted for age, sex, education, and potentially other variables). All effects will be translated into standardized mean difference (Cohen’s d). If needed, the sign of effect sizes will be adjusted so that positive effect sizes reflect greater amyloid burden associated with greater semantic impairment.

To obtain the pooled estimate for each semantic task, random-effects models with inverse variance weighting will be used if a sufficient number of studies (5+) is identified. If only 2-4 studies are identified for a certain semantic task, we will need to use a fixed-effects model with inverse variance weighting for methodological reasons, although we are aware that the yielded results may be too optimistic. A p-value below .05 will be considered as a statistically significant result.

Heterogeneity of the results will be assessed using visual inspection of overlap in confidence intervals in the forest plot, Cochran’s Q test, and I-squared statistic. The amount and impact of between-study variance will be calculated using tau-square.

Differences in the association between amyloid and semantic cognition across the different semantic tasks will be tested by comparing overlap in standardized confidence intervals (Cumming, G. (2009). Inference by eye: reading the overlap of independent confidence intervals. *Statistics in medicine*, *28*(2), 205-220).

The analyses and generation of figures (i.e., forest plots) will be performed in Review Manager (RevMan).

*29. Analysis of subgroups or subsets.*

If significant heterogeneity would be detected, we will look separately at the amyloid assessment method (PET/CSF/blood/histopathology), continuous versus categorical amyloid burden definition, and if a study did or did not control for demographic covariates (e.g., age, sex, education).

*30. Type and method of review.*

- Epidemiologic
- Meta-analysis
- Systematic review

*31. Language.*

English

*32. Country.*

Netherlands

*33. Other registration details.*

N/A

*34. Reference and/or URL for published protocol.*

N/A

*35. Dissemination plans.*

A paper will be submitted to a leading journal in this field.

*36. Keywords.*

Meta-analysis; systematic review; semantic; semantics; biomarker; amyloid; preclinical; cognition; dementia; Alzheimer; Alzheimer’s.

*37. Details of any existing review of the same topic by the same authors.*

This systematic review and meta-analysis is new; the authors have not performed a systematic review or meta-analysis of the same topic before.

*38. Current review status.*

Review_Ongoing

*39. Any additional information.*

N/A

*40. Details of final report/publication(s).*

This field should be left empty until details of the completed review are available.

## Supplementary Text 2. Search strategies in PubMed, Embase, and PsycINFO

### PubMed

(

(

(

(amyloid*[Title/Abstract] OR "Amyloid"[Mesh] OR "Plaque, Amyloid"[Mesh] OR Pittsburgh Compound B[Title/Abstract] OR PIB[Title/Abstract] OR florbetapir[Title/Abstract] OR AV-45[Title/Abstract] OR AV45[Title/Abstract] OR AV1[Title/Abstract] OR AV-1[Title/Abstract] OR florbetaben[Title/Abstract] OR flutemetamol[Title/Abstract])

AND

(PET[Title/Abstract] OR Positron emission tomograph*[Title/Abstract] OR "Positron-Emission Tomography"[Mesh])

)

OR

(

(amyloid*[Title/Abstract] OR "Amyloid"[Mesh] OR "Plaque, Amyloid"[Mesh] OR Abeta40[Title/Abstract] OR Abeta 40[Title/Abstract] OR Abeta42[Title/Abstract] OR Abeta 42[Title/Abstract] OR beta40[Title/Abstract] OR beta 40[Title/Abstract] OR beta42[Title/Abstract] OR beta 42[Title/Abstract] OR Aβ40[Title/Abstract] OR Aβ 40[Title/Abstract] OR Aβ42[Title/Abstract] OR Aβ 42[Title/Abstract] OR β40[Title/Abstract] OR β 40[Title/Abstract] OR β42[Title/Abstract] OR β 42[Title/Abstract])

AND

(CSF[Title/Abstract] OR "Cerebrospinal Fluid"[Mesh] OR cerebrospinal fluid*[Title/Abstract] OR cerebro spinal fluid*[Title/Abstract] OR plasma[Title/Abstract])

)

OR

(

(amyloid*[Title/Abstract] OR "Amyloid"[Mesh] OR "Plaque, Amyloid"[Mesh] OR senile plaques[Title/Abstract])

AND

(neuropatholog*[Title/Abstract] OR amyloid patholog*[Title/Abstract] OR "Neuropathology"[Mesh] OR senile plaques[Title/Abstract])

)

)

AND

(semantic*[Title/Abstract] OR cognition[Title/Abstract] OR cognitive domain*[Title/Abstract] OR cognitive batter*[Title/Abstract] OR cognitive test*[Title/Abstract] OR cognitive performance[Title/Abstract] OR cognitive scor*[Title/Abstract] OR cognitive trajector*[Title/Abstract] OR neuropsychological[Title/Abstract] OR psychometric[Title/Abstract] OR "Semantics"[Mesh] OR "Neuropsychological Tests"[Mesh:NoExp])

AND

(

("Humans"[Mesh] OR individuals[Title/Abstract] OR participants[Title/Abstract] OR older adults[Title/Abstract])

AND

(normal[Title/Abstract] OR nondemented[Title/Abstract] OR non-demented[Title/Abstract] OR without dementia[Title/Abstract] OR aging[Title/Abstract] OR ageing[Title/Abstract] OR aged[Title/Abstract] OR elderly[Title/Abstract] OR "Prodromal Symptoms"[Mesh] OR "Aged"[Mesh] OR older[Title/Abstract] OR preclinical[Title/Abstract] OR pre-clinical[Title/Abstract] OR unimpaired[Title/Abstract])

)

)

### Embase

(

(

(

(amyloid*:ti,ab,kw OR ‘Amyloid’/exp OR 'amyloid plaque'/exp OR ‘Pittsburgh Compound B’:ti,ab,kw OR PIB:ti,ab,kw OR florbetapir:ti,ab,kw OR ‘AV-45’:ti,ab,kw OR AV45:ti,ab,kw OR AV1:ti,ab,kw OR ‘AV-1’:ti,ab,kw OR florbetaben:ti,ab,kw OR flutemetamol:ti,ab,kw)

AND

(PET:ti,ab,kw OR ‘Positron emission tomograph*’:ti,ab,kw OR ‘positron emission tomography’/exp)

)

OR

(

(amyloid*:ti,ab,kw OR ‘Amyloid’/exp OR 'amyloid plaque'/exp OR Abeta40:ti,ab,kw OR ‘Abeta 40’:ti,ab,kw OR Abeta42:ti,ab,kw OR ‘Abeta 42’:ti,ab,kw OR beta40:ti,ab,kw OR ‘beta 40’:ti,ab,kw OR beta42:ti,ab,kw OR ‘beta 42’:ti,ab,kw OR Aβ40:ti,ab,kw OR ‘Aβ 40’:ti,ab,kw OR Aβ42:ti,ab,kw OR ‘Aβ 42’:ti,ab,kw OR β40:ti,ab,kw OR ‘β 40’:ti,ab,kw OR β42:ti,ab,kw OR ‘β 42’:ti,ab,kw)

AND

(CSF:ti,ab,kw OR ‘Cerebrospinal Fluid’/exp OR ‘cerebrospinal fluid*’:ti,ab,kw OR ‘cerebro spinal fluid*’:ti,ab,kw OR plasma:ti,ab,kw)

)

OR

(

(amyloid*:ti,ab,kw OR ‘Amyloid’/exp OR 'amyloid plaque'/exp OR ‘senile plaques’:ti,ab,kw)

AND

(neuropatholog*:ti,ab,kw OR ‘amyloid patholog*’:ti,ab,kw OR ‘Neuropathology’/exp OR ‘senile plaques’:ti,ab,kw)

)

)

AND

(semantic*:ti,ab,kw OR cognition:ti,ab,kw OR ‘cognitive domain*’:ti,ab,kw OR ‘cognitive batter*’:ti,ab,kw OR ‘cognitive test*’:ti,ab,kw OR ‘cognitive performance’:ti,ab,kw OR ‘cognitive scor*’:ti,ab,kw OR ‘cognitive trajector*’:ti,ab,kw OR neuropsychological:ti,ab,kw OR psychometric:ti,ab,kw OR ‘Semantic memory’/exp OR ‘Semantics’/exp OR ‘Neuropsychological Test’/de)

AND

(

(‘Human’/exp OR individuals:ti,ab,kw OR participants:ti,ab,kw OR ‘older adults’:ti,ab,kw)

AND

(normal:ti,ab,kw OR nondemented:ti,ab,kw OR ‘non-demented’:ti,ab,kw OR ‘without dementia’:ti,ab,kw OR aging:ti,ab,kw OR ageing:ti,ab,kw OR aged:ti,ab,kw OR elderly:ti,ab,kw OR ‘Prodromal Symptom’/exp OR ‘Aged’/exp OR older:ti,ab,kw OR preclinical:ti,ab,kw OR ‘pre-clinical’:ti,ab,kw OR unimpaired:ti,ab,kw)

)

)

NOT 'conference abstract'/it

### PsycINFO

(

(

(

(amyloid*.ab,ti. OR exp Beta Amyloid/ OR exp Amyloid Precursor Protein/ OR Pittsburgh Compound B.ab,ti. OR PIB.ab,ti. OR florbetapir.ab,ti. OR AV-45.ab,ti. OR AV45.ab,ti. OR AV1.ab,ti. OR AV-1.ab,ti. OR florbetaben.ab,ti. OR flutemetamol.ab,ti.)

AND

(PET.ab,ti. OR Positron emission tomograph*.ab,ti. OR exp Positron Emission Tomography/)

)

OR

(

(amyloid*.ab,ti. OR exp Beta Amyloid/ OR exp Amyloid Precursor Protein/ OR Abeta40.ab,ti. OR Abeta 40.ab,ti. OR Abeta42.ab,ti. OR Abeta 42.ab,ti. OR beta40.ab,ti. OR beta 40.ab,ti. OR beta42.ab,ti. OR beta 42.ab,ti.)

AND

(CSF.ab,ti. OR exp Cerebrospinal Fluid/ OR cerebrospinal fluid*.ab,ti. OR cerebro spinal fluid*.ab,ti. OR plasma.ab,ti.)

)

OR

(

(amyloid*.ab,ti. OR exp Beta Amyloid/ OR exp Amyloid Precursor Protein/ OR senile plaques.ab,ti. OR exp Senile Plaques/)

AND

(neuropatholog*.ab,ti. OR amyloid patholog*.ab,ti. OR exp Neuropathology/ OR senile plaques.ab,ti.)

)

)

AND

(semantic*.ab,ti. OR cognition.ab,ti. OR cognitive domain*.ab,ti. OR cognitive batter*.ab,ti. OR cognitive test*.ab,ti. OR cognitive performance.ab,ti. OR cognitive scor*.ab,ti. OR cognitive trajector*.ab,ti. OR neuropsychological.ab,ti. OR psychometric.ab,ti. OR exp Semantics/ OR exp Semantic Memory/ OR exp Neuropsychology/ OR exp Psychometrics/ OR exp Neuropsychological Assessment/ OR exp Test Performance/ OR exp Cognitive Ability/ OR exp Cognitive Impairment/ OR exp Performance Tests/ OR exp Cognition/)

AND

(

(human.po. OR individuals.ab,ti. OR participants.ab,ti. OR older adults.ab,ti.)

AND

(normal.ab,ti. OR nondemented.ab,ti. OR non-demented.ab,ti. OR without dementia.ab,ti. OR aging.ab,ti. OR ageing.ab,ti. OR aged.ab,ti. OR elderly.ab,ti. OR exp aging/ OR older.ab,ti. OR preclinical.ab,ti. OR pre-clinical.ab,ti. OR unimpaired.ab,ti.)

)

)

## Supplementary Text 3. Adjusted version of the Newcastle-Ottawa Quality Assessment Scale Cohort Studies

Note: A study can be awarded a maximum of one star for each numbered item within the Selection and Outcome categories. A maximum of four stars can be given for Comparability. Total maximum number of stars is nine.

**Selection**

1. Representativeness of the exposed cohort (amyloid positive)
   1. truly representative of the average older adult without dementia in the community (i.e., community based cohort and mix of non-demented individuals with and without subjective complaints, can include individuals with MCI as well) 🟋
   2. somewhat representative of the average older adult without dementia in the community (e.g., if a certain selection is made which makes the individuals 'more' cognitively normal, e.g., only non-demented individuals without subjective complaints or only non-demented individuals without MCI) 🟋
   3. selected group of users, e.g., volunteers, memory clinic visitors, only individuals at higher risk (only subjective complaints, only depressive sypmtoms, only APOE e4 carriers)
   4. no description of the derivation of the cohort
2. Selection of the non-exposed cohort (amyloid negative)
   1. drawn from the same community as the exposed cohort 🟋
   2. drawn from a different source
   3. no description of the derivation of the non-exposed cohort
3. Ascertainment of exposure
   1. continuous measurement 🟋
   2. categorized based on established or published cut-offs 🟋
   3. categorized based on non-established cut-offs (e.g., z-score cut-off, mean split, median split)
   4. no description

**Comparability**

1. Comparability of cohorts on the basis of the design or analysis
   - study controls for age 🟋
   - study controls for sex/gender 🟋
   - study controls for education 🟋
   - study controls for any additional factor 🟋

**Outcome**

1. Ascertainment of outcome
   1. independent neuropsychological assessment 🟋
   2. record linkage
   3. self-report
   4. no description
2. Same method of assessment for cases (amyloid positive) and controls (amyloid negative)
   1. yes 🟋
   2. no

## Supplementary Figures. Forest plots for subgroup analyses

Supplementary Figure 1. Forest plot picture naming for subgroups by subjective cognitive impairment sample selection


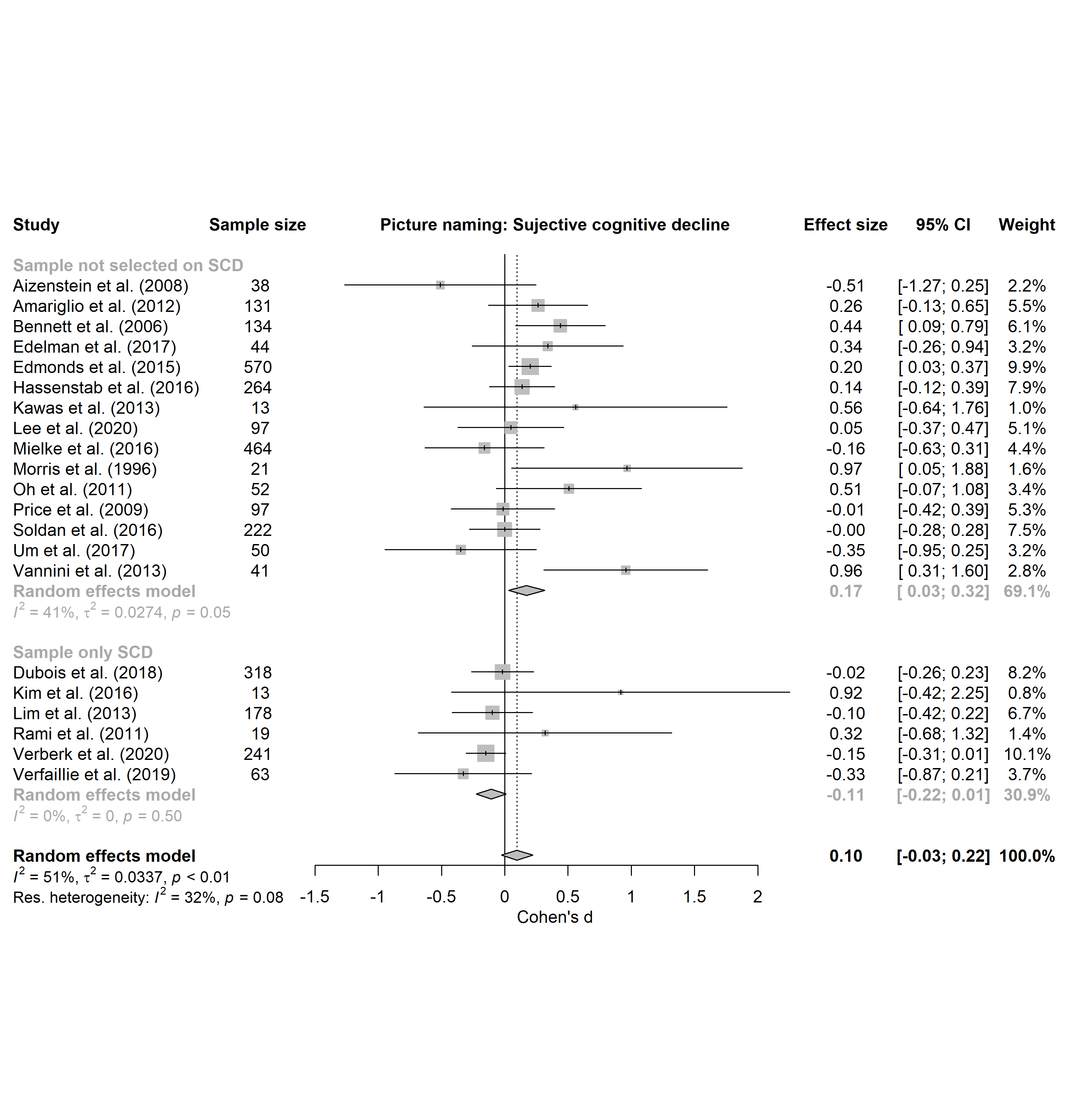


Supplementary Figure 2. Forest plot picture naming for subgroups by covariate adjustment


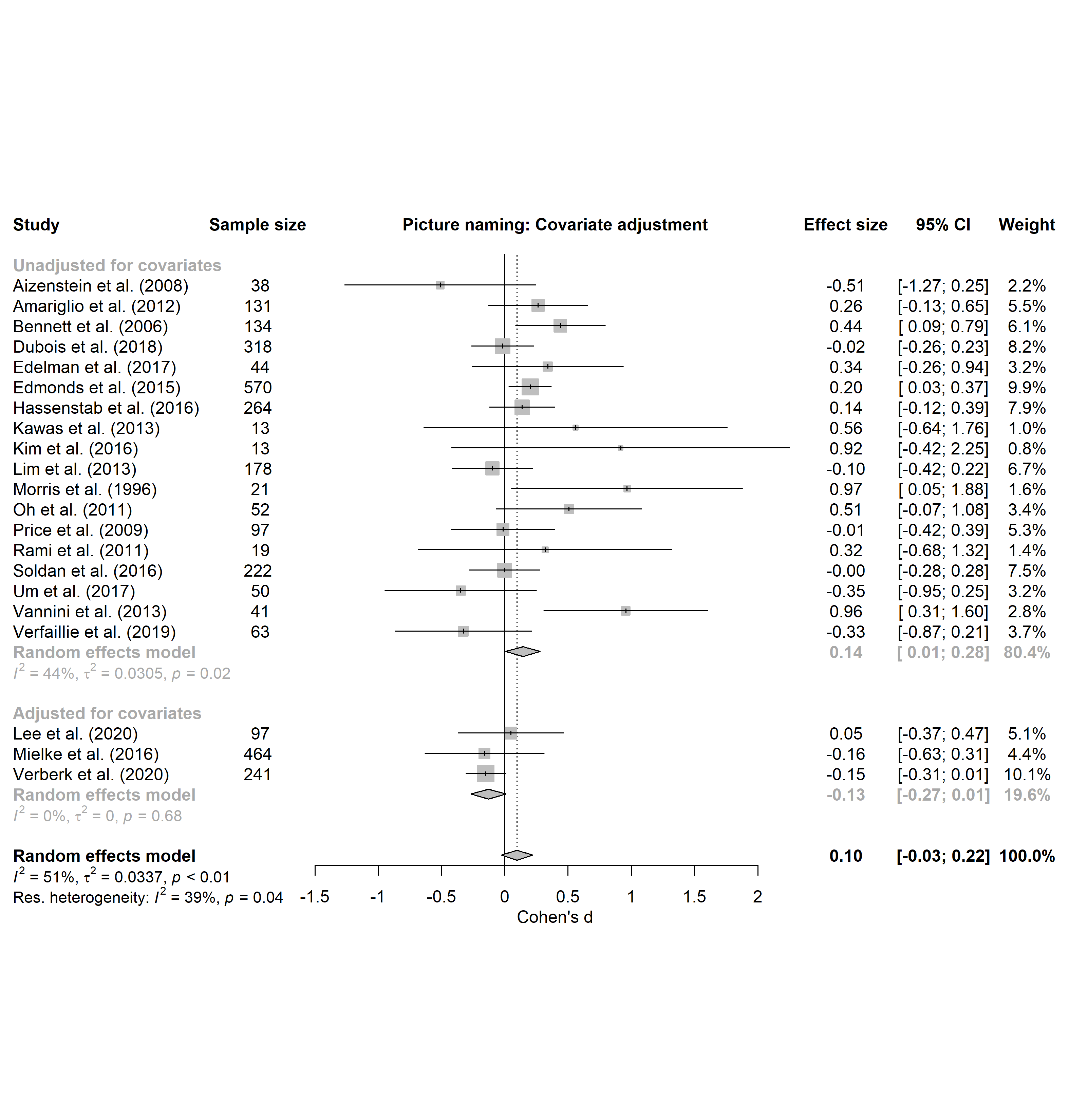


Supplementary Figure 3. Forest plot picture naming for subgroups by sample mean age


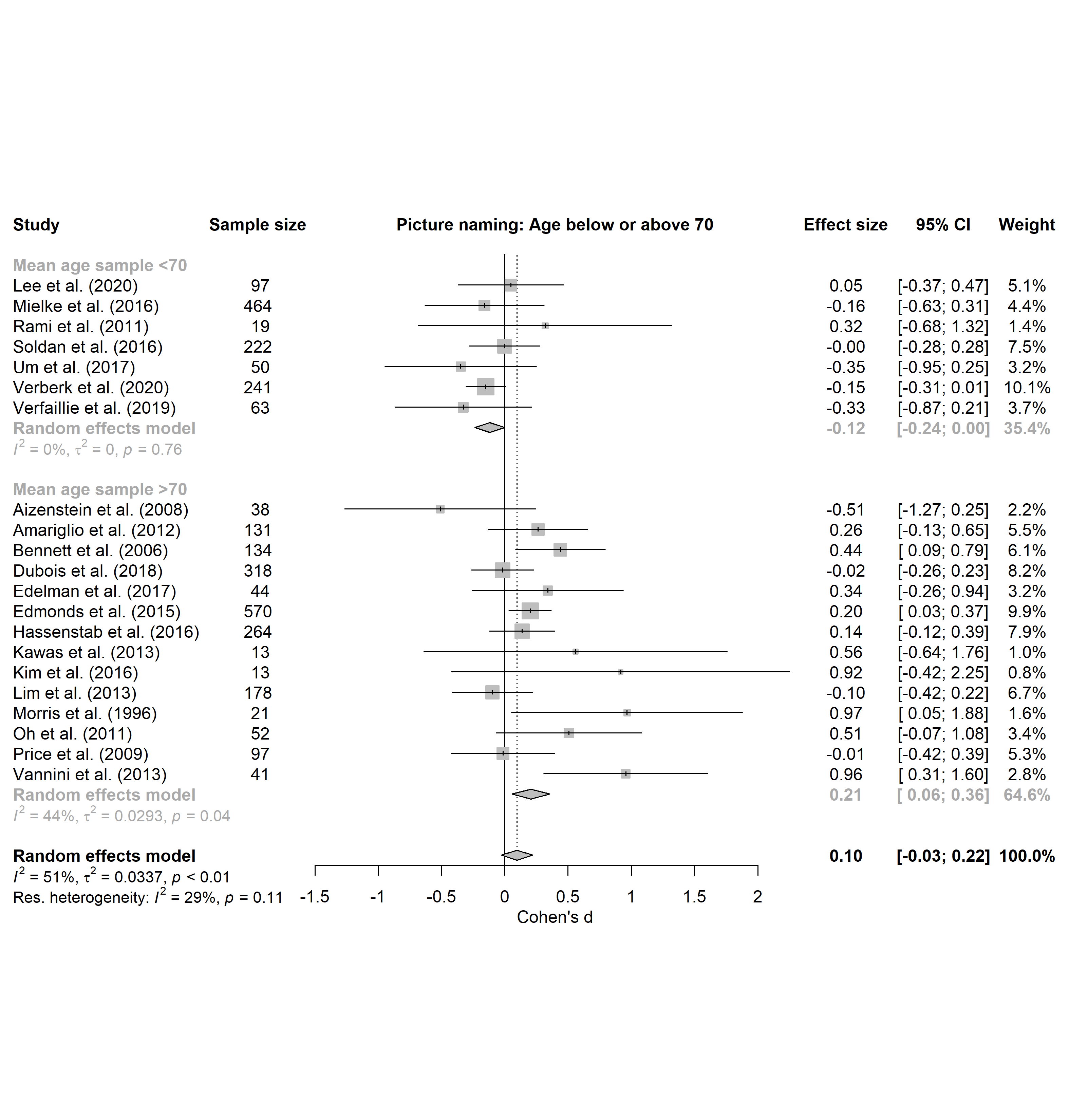


Supplementary Figure 4. Forest plot picture naming for subgroups by amyloid scale


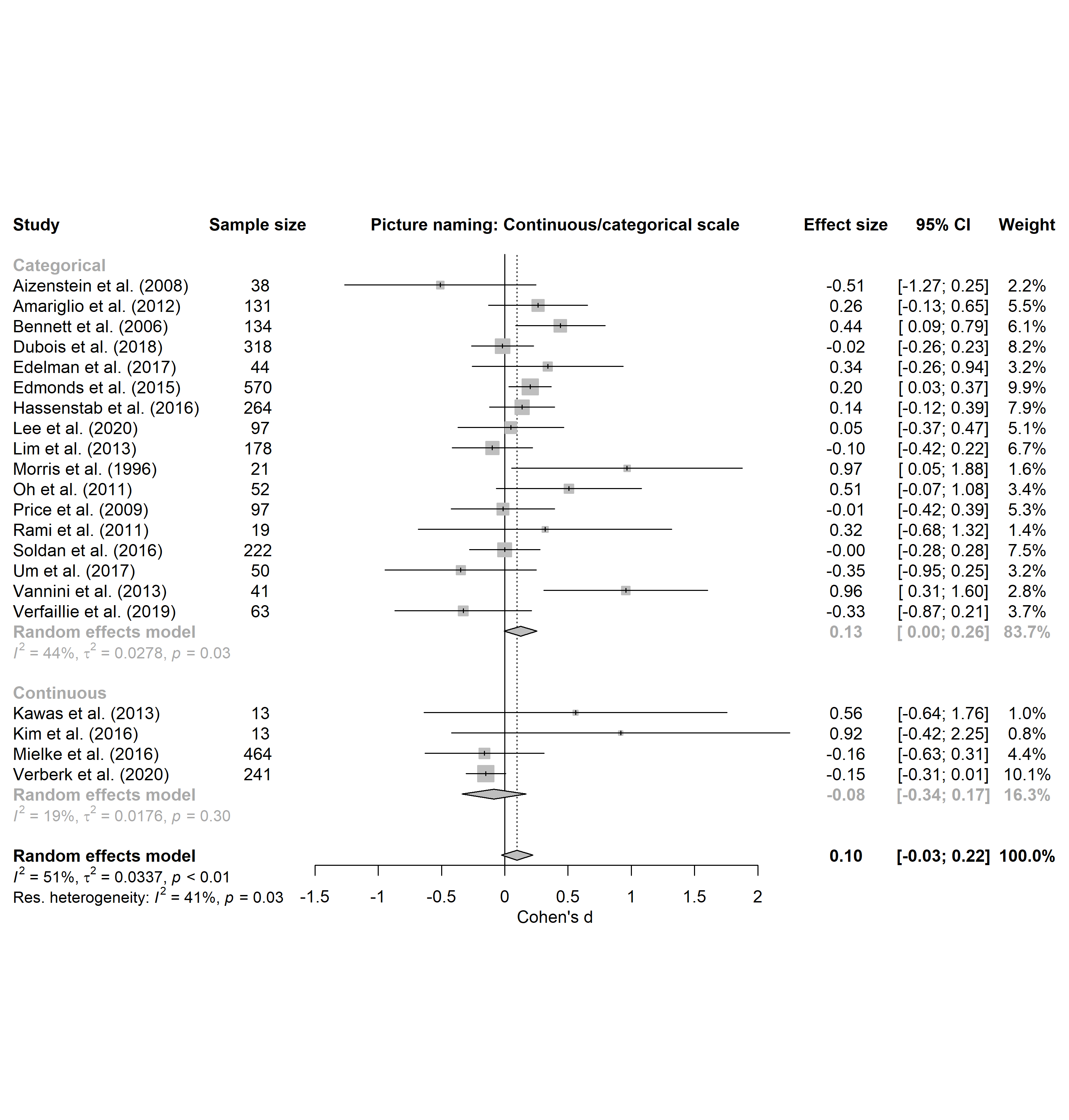


Supplementary Figure 5. Forest plot picture naming for subgroups by amyloid measure


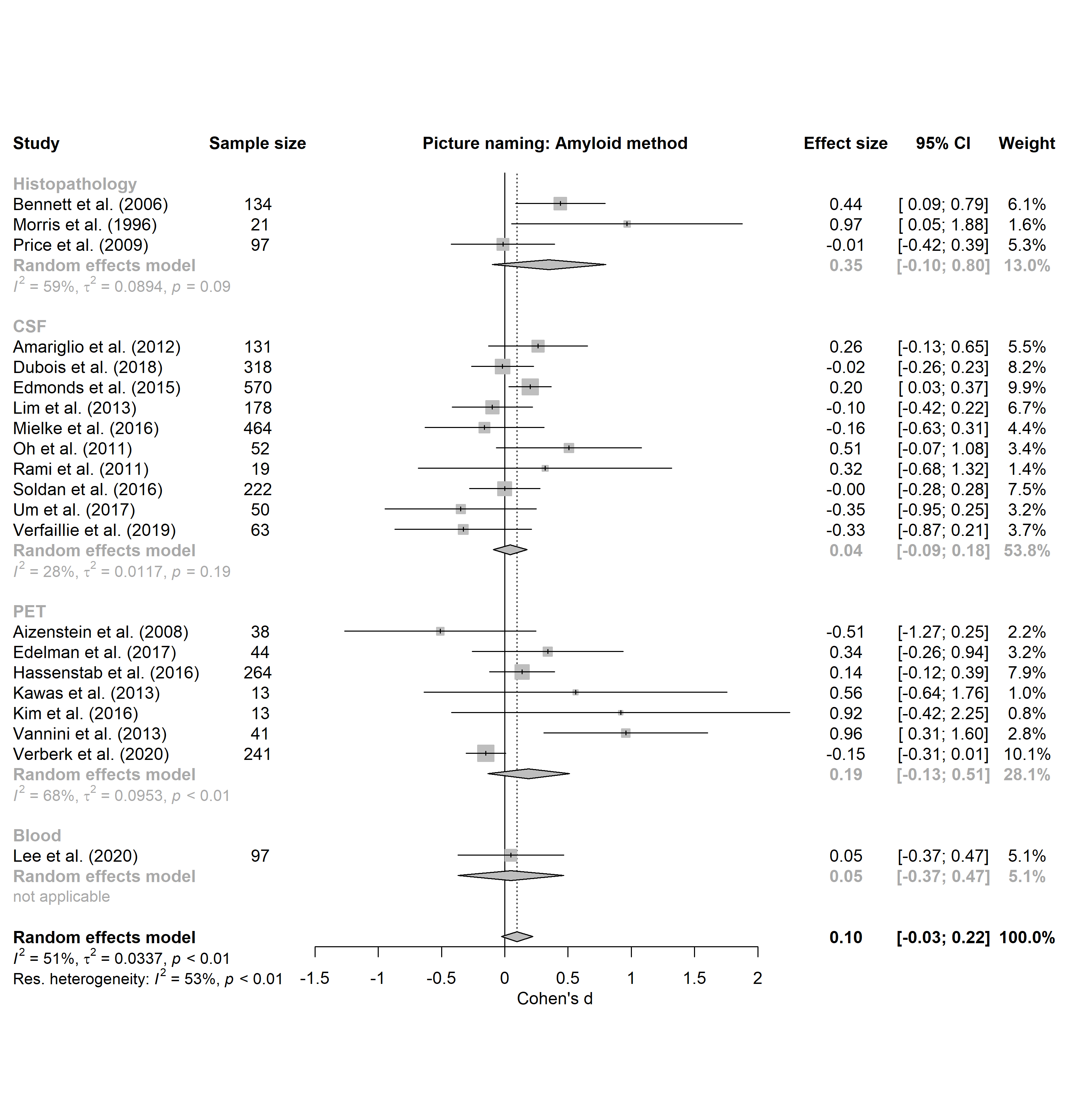


Supplementary Figure 6. Forest plot semantic fluency for subgroups by subjective cognitive impairment sample selection


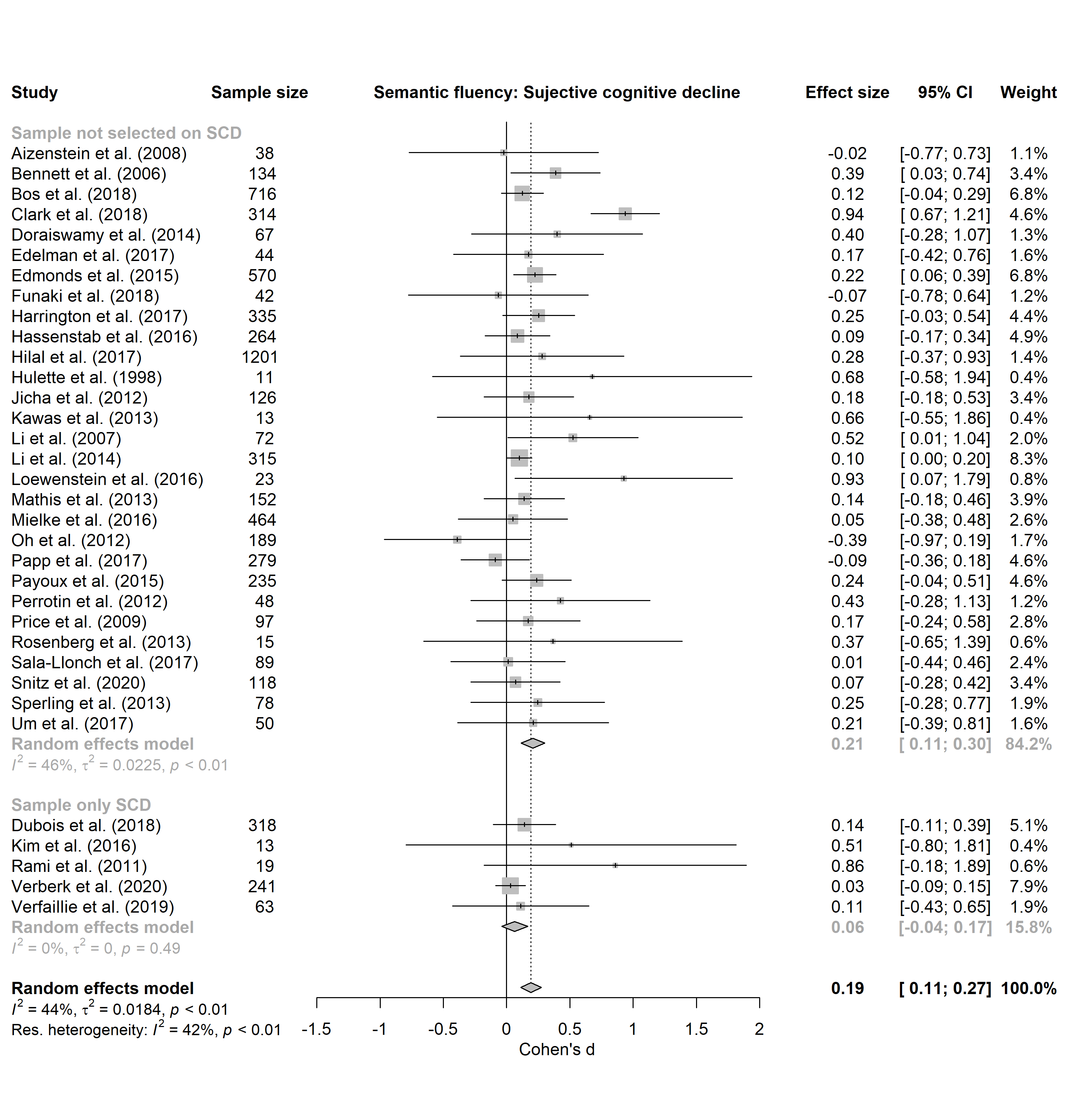


Supplementary Figure 7. Forest plot semantic fluency for subgroups by covariate adjustment


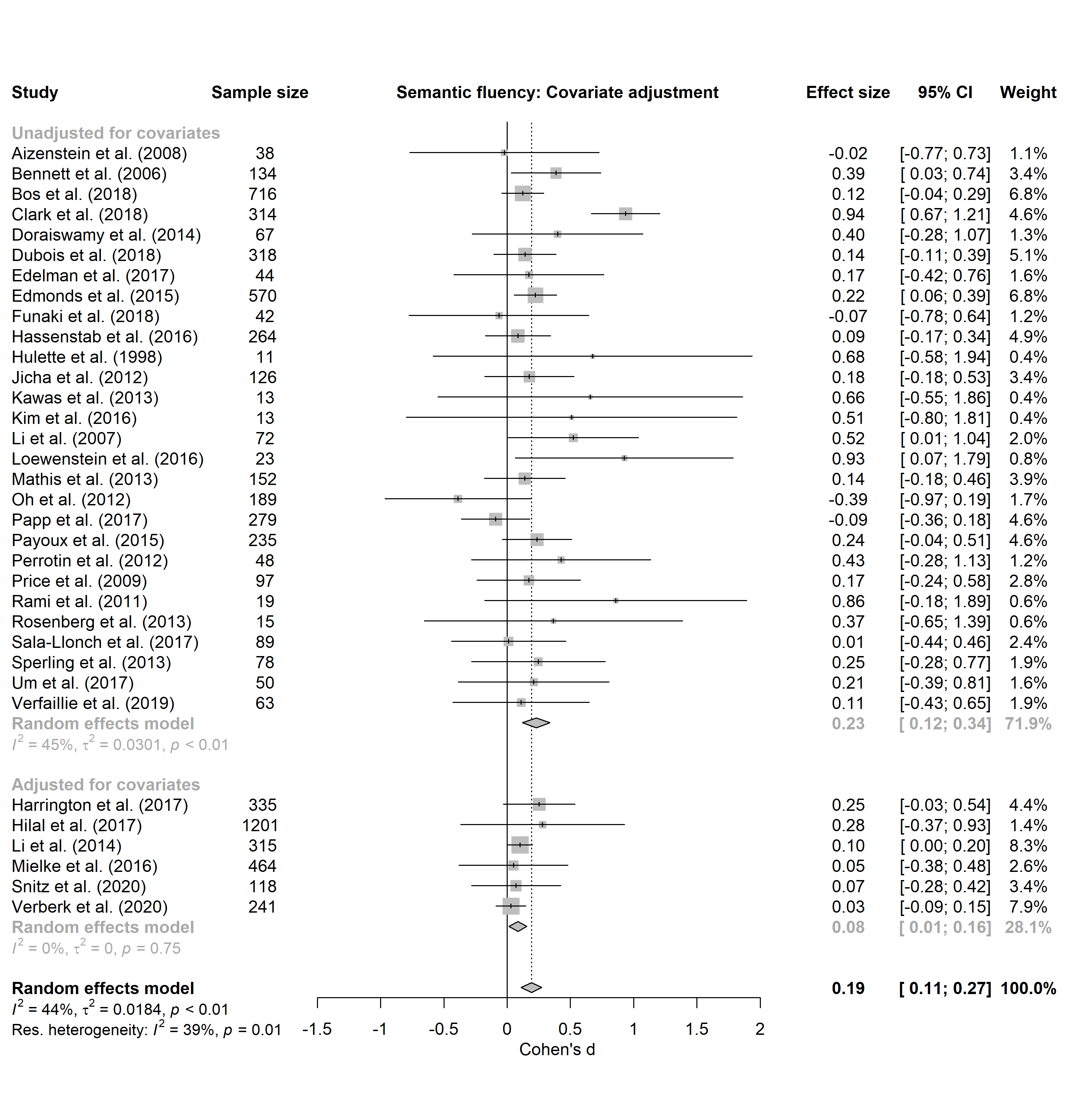


Supplementary Figure 8. Forest plot semantic fluency for subgroups by sample mean age


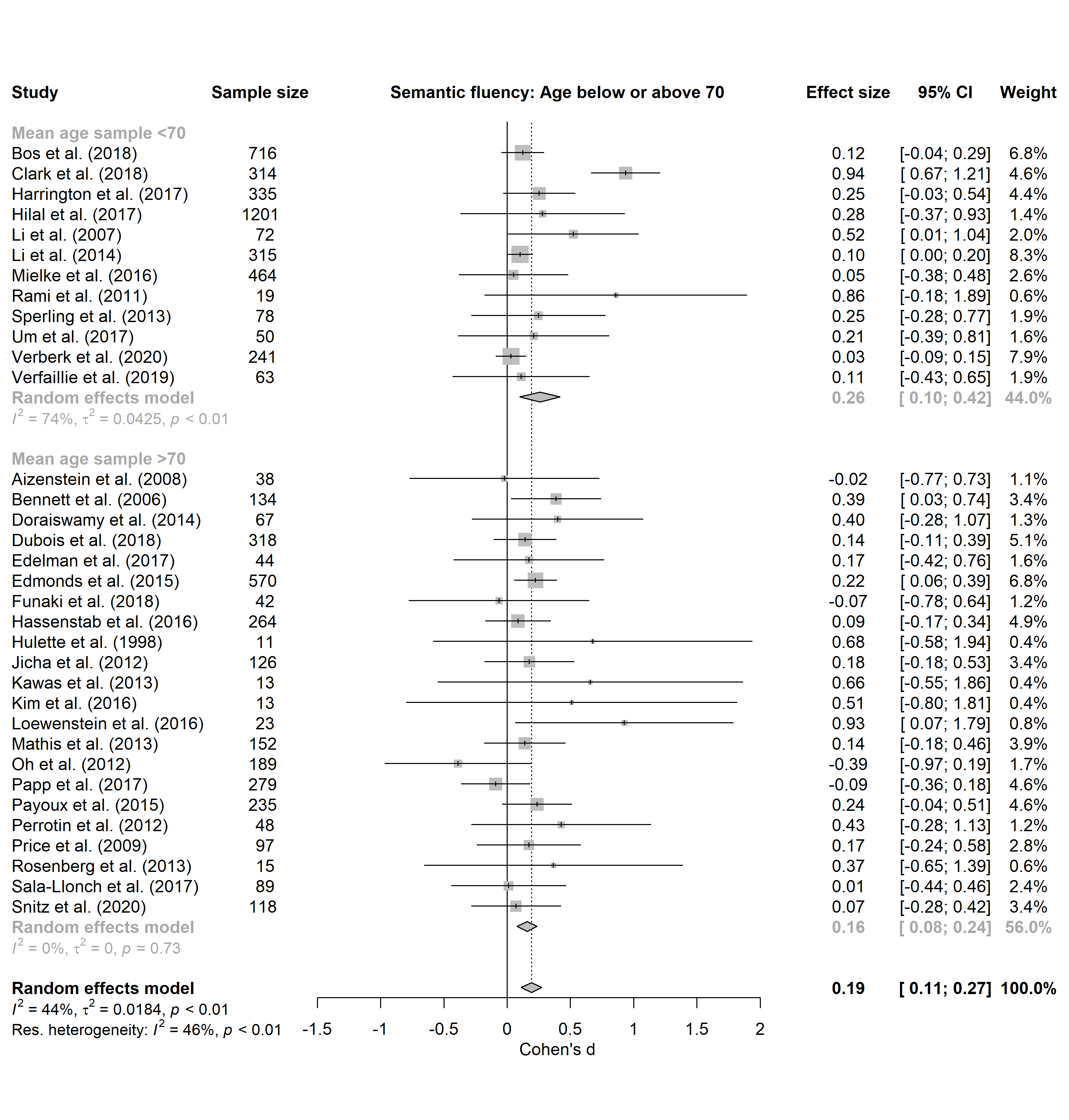


Supplementary Figure 9. Forest plot semantic fluency for subgroups by amyloid scale


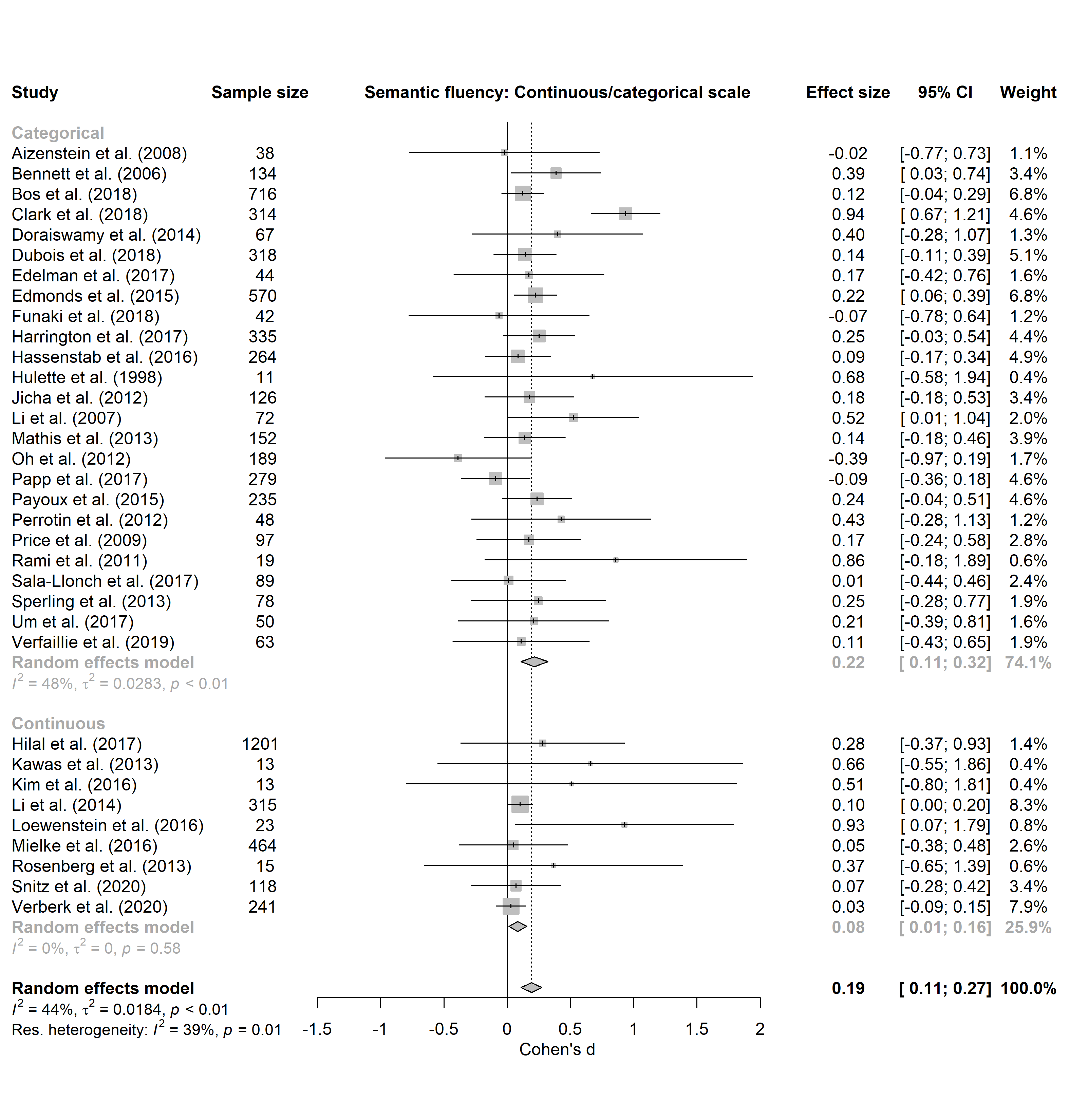


Supplementary Figure 10. Forest plot semantic fluency for subgroups by amyloid measure


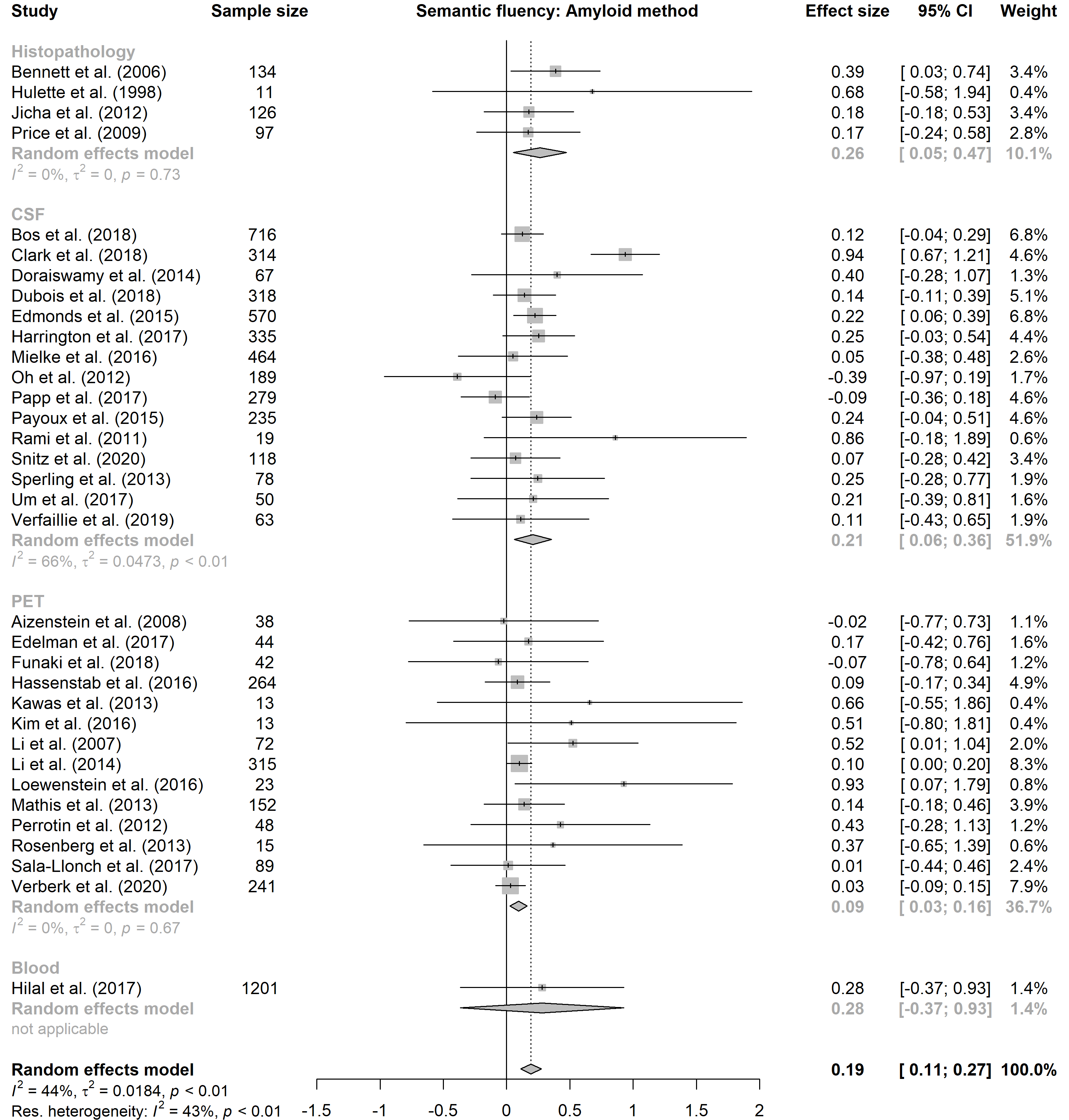

Supplement: Supplementary Materials [file NIHMS1677309-supplement-Supplementary_Materials.docx]
